# DIDS modulates VDAC1 oligomerization to suppress intrinsic apoptosis and attenuates *in vitro* and *in vivo* RSV infection

**DOI:** 10.1128/jvi.02200-25

**Published:** 2026-02-11

**Authors:** Siyu Lin, Xiaotong Chen, Meihua Luo, Xiaolu Cui, You Dai, Zhen Sun, Guikang Wang, Hong Peng, Ping Ling, Jinlin Long, Huifang Zhou, Changlei Luo, Yan-Fei Qi, Ke Zhang, Yu-Si Luo

**Affiliations:** 1 Guizhou Key Laboratory of Microbio and Infectious Disease Prevention & Control, Institute of Virology, The Key and Characteristic Laboratory of Modern Pathogenicity Biology, School of Basic Medicine, Guizhou Medical University 650838, Guiyang, Guizhou, China; 2 School of Basic Medical Sciences, Guangzhou Medical University, Guangzhou National Laboratory 655509, Guangzhou, Guangdong, China; 3 Emergency Department, The Affiliated Hospital of Guizhou Medical University 74720, Guiyang, Guizhou, China; 4 PICU, Guiyang Maternal and Child Health Care Hospital, Guiyang, Guizhou, China; 5 Department of Pharmacology, College of Osteopathic Medicine, Duquesne University 6613, Pittsburgh, Pennsylvania, USA; 6 Department of Emergency, Liupanshui Hospital of The Affiliated Hospital of Guizhou Medical University, Liupanshui, Guizhou, China; Loyola University Chicago - Health Sciences Campus, Maywood, Illinois, USA

**Keywords:** RSV, DIDS, VDAC1, oligomerization, apoptosis, *in vitro* and *in vivo* antivirus

## Abstract

Human respiratory syncytial virus (RSV) is a major pathogen causing acute lower respiratory tract infections in infants, young children, and elderly people worldwide. Viruses often hijack host cell ion channels to optimize their intracellular environment, positioning ion channel blockers as promising antiviral agents. On the outer mitochondrial membrane, voltage-dependent anion channel protein 1 (VDAC1) plays a crucial role in regulating mitochondrial pathway apoptosis and maintaining cellular homeostasis. This study systematically evaluates the antiviral activity of the VDAC1 inhibitor 4,4′-diisothiocyanatostilbene-2,2′-disulfonic acid (DIDS), both *in vitro* and *in vivo*. The results demonstrate that VDAC1 is a key factor in RSV infection, and DIDS significantly inhibits viral replication. Functional intervention experiments show that DIDS effectively blocks RSV-induced VDAC1 oligomerization in the mitochondrial membrane, suppressing mitochondrial apoptosis and disrupting chloride ion (Cl^−^) flux, thereby inhibiting viral replication. Exogenous Cl^−^ supplementation reverses these effects, further highlighting the critical role of VDAC1 in the life cycle of RSV. In conclusion, the antiviral effects and mechanistic insights of DIDS reveal that VDAC1 regulates mitochondrial-mediated apoptosis while also modulating anion homeostasis to promote viral replication. These findings provide a potential target and theoretical foundation for the development of novel antiviral strategies targeting mitochondrial ion channels.

**IMPORTANCE:**

The study evaluates the antiviral activity of the VDAC1 inhibitor DIDS against respiratory syncytial virus (RSV), a major cause of lower respiratory tract infections worldwide. VDAC1 is identified as a key factor in RSV infection, with DIDS significantly inhibiting viral replication by blocking RSV-induced VDAC1 oligomerization in the mitochondrial membrane, suppressing mitochondrial apoptosis, and disrupting chloride ion flux. These findings establish that VDAC1-mediated regulation of anion homeostasis and subsequent mitochondrial-mediated apoptosis is a critical mechanism promoting RSV replication, providing a novel target for antiviral strategies.

## INTRODUCTION

Human respiratory syncytial virus (RSV) is a leading cause of severe lower respiratory tract infections in infants, young children, elderly people, and immunodeficient adults, accounting for substantial morbidity and mortality worldwide (1, 2). Although recent advances in vaccines and antiviral agents represent important progress, their clinical efficacy remains limited, and the continual emergence of viral variants poses a major challenge (3). Given the virus’s strong reliance on host cellular machinery, host-directed therapeutics are gaining traction as an alternative strategy (4). Unlike direct-acting antivirals, host-targeted approaches present a higher genetic barrier to resistance, as viral escape through genomic mutation is considerably more constrained (5).

Ion channels have emerged as attractive host targets for antiviral intervention (6). These pore-forming proteins control the selective transport of ions such as Ca²^+^, Cl^−^, K^+^, and Na^+^ across plasma and organellar membranes, thereby shaping essential processes, including cellular apoptosis, metabolism, and signal transduction (7). Accumulated evidence indicates numerous viruses exploit host ion channel activity to optimize their replication cycles (8). Proof-of-concept studies with established ion channel inhibitors support this observation; for instance, our group recently verified the well-defined Ca²^+^ channel blocker Verapamil-attenuated RSV infection (9), while other researchers observed chloride channel antagonists alleviated rotavirus-induced diarrhea (10–12). Collectively, such findings highlight the therapeutic promise of ion channel–based antiviral strategies.

The voltage-dependent anion channel 1 (VDAC1), a highly conserved protein mainly located in the mitochondrial outer membrane, serves as a critical gateway for the exchange of metabolites and negative ions, playing a central role in maintaining metabolic homeostasis and regulating mitochondrial pathway apoptosis (13). Under apoptotic stimulation, VDAC1 can oligomerize to form large pores, leading to increased mitochondrial membrane permeability and the release of apoptogenic factors and anions (such as Cl^−^), thereby triggering programmed cell death (14). Previous studies have reported several viruses, including influenza and dengue virus, interact directly or indirectly with VDAC1 to facilitate their replication (15). Recent multi-omics analyses have also suggested an association between RSV infection and altered VDAC1 expression (16). However, how RSV functionally depends on VDAC1, and through what precise mechanisms VDAC1 modulates both apoptotic pathways and viral replication, remains obscure. The 4, 4′-diisothiocyanatostilbene-2, 2′-disulfonic acid (DIDS) is a small-molecule compound that effectively inhibits VDAC1, blocking its oligomerization and the subsequent apoptosis that the oligomerization induces (17). Although a previous study has indicated that DIDS inhibits RSV infection in A549 cells, the underlying mechanism remains unclear (18).

In the current study, we investigated the *in vitro* and *in vivo* anti-RSV activity of DIDS, specifically by examining the role of VDAC1.

## MATERIALS AND METHODS

### Cells, virus, and drugs

Human laryngeal epithelial carcinoma cells (HEp-2) and A549 cells (CCL23 and CCL181, ATCC, Manassas, VA, USA) were cultured in DMEM/F12-GlutaMAX medium (10565, Thermo Fisher Scientific Inc., Grand Island, NY, USA) supplemented with 5% fetal bovine serum (FBS, 10270106, Thermo Fisher Scientific Inc., Grand Island, NY, USA) and 1% penicillin-streptomycin (P/S, 15140122, Thermo Fisher Scientific Inc., Grand Island, NY, USA) for HEp-2 cells, and in DMEM medium (11965, Thermo Fisher Scientific Inc., Grand Island, NY, USA) supplemented with 7% FBS and 1% P/S for A549 cells. HEp-2 and A549 cells were all cultured at 37°C with 5% CO_2_.

The RSV strain used in this study was the mouse-adapted highly pathogenic strain (GZ08-18), which was derived through serial passage of the GZ08-0 strain in 8-month-old female BALB/c mice by our group (19, 20). Briefly, the GZ08-0 strain was clinically isolated from Guangzhou Children’s Hospital, Guangzhou, China, in 2008 and generously gifted by Prof. Qiwei Zhang, Jinan University, China. GZ08-0 and GZ08-18 were sequenced, and complete genomes were uploaded into GenBank with accession nos. KP218910 and KP119747, respectively (21). GZ08-18 was propagated in HEp-2 cells using DMEM/F12-GlutaMAX medium supplemented with 2% FBS.

The 4,4′-diisothiocyanato-2,2′-biphenylsulfonic acid disodium salt (DIDS, CAS no., 67483-13-0, HY-D0086, purity = 99.13%), Ribavirin (36791-04-5, HY-B0434, purity = 98.00%), Rotenone (83-79-4, HY-B1756, purity = 99.74%), Z-VAD-FMK (161401-82-7, HY-16658B, purity = 99.78%), potassium chloride (KCl, 7447-40-7, HY-Y0537, purity = 99.50%), calcium chloride (CaCl_2_, 10043-52-4, HY-Y0406H, purity = 98.20%), and EGTA (67-42-5, HY-D0861, purity = 98.0%) were offered from MedChemExpress Co., Ltd., Shanghai, China. The 3-(4,5-dimethylthiazol-2-yl)−2,5-diphenyltetrazolium bromide (MTT, 298-93-1, M8180, Solarbio Life Science, Beijing, China) was used for cell viability assay.

### Plasmid and siRNAs

The human VDAC1 coding sequence (GenBank accession no. NM_003374.3) was cloned into the pcDNA3.1 vector to construct a VDAC1-overexpression plasmid with a C-terminal Flag tag named pcDNA3.1-VDAC1. Four small interfering RNAs (siRNAs) specifically targeted to VDAC1 (*si-VDAC1-1173, si-VDAC1-1280, si-VDAC1-1686,* and *si-VDAC1-1778*) and a non-targeting siRNA control (*siRNA-NC*) were designated and synthesized by GenePharma, Shanghai, China, with the sequences provided in Table S1.

### Antibodies and reagents

The primary antibodies used for Western blotting were as follows: mouse anti-VDAC1 (ab186321, Abcam, Shanghai, China, diluted to 1:1,000), mouse anti-BCL-2 (12789-1-AP, Proteintech, Wuhan, China, 1:5,000), rabbit anti-GAPDH (60004-1-Ig, Proteintech, Wuhan, China, 1:5,000), and rabbit anti-β-actin (AC048, ABclonal, Wuhan, China, 1:10,000). For secondary antibodies in Western blotting, HRP-conjugated goat anti-rabbit IgG (GB23303-1, Servicebio, Wuhan, China, 1:5,000) and HRP-conjugated goat anti-mouse IgG (SA00001-1, Proteintech, Wuhan, China, 1:3,000) were utilized. For immunofluorescence assays, the primary antibodies included rabbit anti-VDAC1 (A19707, ABclonal, Wuhan, China, 1:100), mouse anti-RSV fusion (F) protein (sc-101362, Santa Cruz Biotechnology, Dallas, TX, USA, 1:100), and rabbit anti-TOM20 (11802-1-AP, Proteintech, Wuhan, China, 1:500). The corresponding secondary antibodies were Alexa Fluor 488- or Alexa Fluor 594-conjugated goat anti-mouse or anti-rabbit IgG (ab150113, 1:300; ab150080, 1:200), purchased from Abcam, Shanghai, China.

Lipofectamine 3000 (L3000015, Thermo Fisher Scientific, Grand Island, NY, USA) and GP-Transfection-Mate (G04008, GenePharma, Suzhou, China) were used for plasmid and siRNA transfections, respectively. The SuperSignal ECL substrate kit (36208ES60, YEASEN, Shanghai, China) and ethylene glycol bis (succinimidyl succinate, EGS; 21024ES60, YEASEN, Shanghai, China) were used for protein expression detection and crosslinking.

Cytokine levels were quantified using ELISA kits for mouse TNF-α (EK282), IFN-γ (EK280), and IL-1β (EK201BHS) purchased from MultiSciences (Lianke) Biotech, Hangzhou, China.

### *In vitro* experiments

#### Cytotoxicity detection assay

The cytotoxicity of DIDS in HEp-2 and A549 cells was determined by MTT assay. Cells were seeded and treated with serial dilutions of DIDS for 48 h post-infection (hpi). The medium was then replaced with MTT solution and incubated for 4 h. Formazan crystals were dissolved in DMSO, and absorbance at 570 nm was measured using the microplate reader (ELx808, BioTek Instruments, Inc., Winooski, VT, USA). Concentration–response curves were fitted, and the coefficient of determination (*R*²) was verified to exceed 0.95. The half-maximal cytotoxic concentration (CC_50_) was calculated accordingly.

#### *In vitro* antiviral assay

HEp-2 and A549 cells were pre-seeded in 96-well plates and infected with GZ08-18 at a multiplicity of infection (MOI) of 0.1 for 1 h at 37°C and 5% CO_2_. After removing the inoculum, cells were overlaid with medium containing either 50 µM Ribavirin (positive control) or graded concentrations of DIDS. At 48 hpi, culture supernatant samples were harvested for the TCID_50_ assay. Concentration–response curves were fitted, and the *R*² was verified to exceed 0.95. The half-maximal inhibitory concentration (IC_50_) was calculated accordingly. The Selectivity Index (SI) of DIDS against RSV infection in HEp-2 or A549 cells was deduced as SI = CC_50_ / IC_50_.

#### VDAC1 silencing and overexpressing experiments

For the VDAC1 silencing experiment, pre-seeded HEp-2 cells were transfected with siRNAs-involved GP-Transfection-Mate in Opti-MEM (31985070, Thermo Fisher Scientific Inc., Grand Island, NY, USA) for 24 h. Knockdown efficiency was confirmed by immunoblotting (Fig. S3). After confirming that 80 nM *siRNA-NC* had no toxic effect on the cells, *si-VDAC1-1778* was selected from four siRNAs due to its optimal gene silencing effect. In subsequent experiments, HEp-2 cells were transfected with 20, 40, and 80 nM *si-VDAC1-1778* for 24 h, followed by 0.1 MOI RSV infection for 1 h. Then the inoculum was replaced with DMEM/F12-GlutaMAX, and supernatant samples were collected at 48 hpi for the TCID_50_ assay.

For the VDAC1 overexpression experiment, pre-seeded HEp-2 cells were transfected with 2.5 µg of pcDNA3.1-VDAC1 per well in Lipofectamine 3000 for 24 h. An empty vector (Vehicle) transfection group and a non-transfected (Control) group were included as negative controls. At 48 h post-transfection, cells were harvested, and total protein was extracted using pre-cooled RIPA lysis buffer supplemented with protease inhibitors. The protein concentration was determined using a BCA Protein Assay Kit (P0012, Beyotime, Beijing, China). Equal amounts of protein were separated by SDS-PAGE, and Western blotting (WB) was performed using antibodies against VDAC1 and β-actin to confirm the overexpression of VDAC1. Subsequently, the VDAC1-overexpressing cells and corresponding control cells were infected with GZ08-18 at an MOI of 0.1. After 48 hpi, the WB assay was repeated to re-validate the sustained overexpression of VDAC1 and assess relevant protein level changes. Meanwhile, the culture supernatants were harvested to quantify RSV replication using the TCID_50_ assay.

### Time-of-addition assay

Pre-seeded HEp-2 cells were infected with 0.05 MOI GZ08-18 for 1 h at 37°C in 5% CO_2_. After removing the inoculum, cells were overlaid with DMEM/F12-GlutaMAX medium. Different concentrations of DIDS were added to the medium at 1, 12, and 24 hpi. In a separate experiment, RSV and different concentrations of DIDS were pre-incubated at 37°C for 1 h before being added to HEp-2 cells. Supernatant samples were collected 48 hpi for viral titration by TCID_50_ assay.

### WB assay

Cells were extracted with lysing in pre-cool RIPA lysis buffer (P0013B, Beyotime, Beijing, China, containing protease inhibitor [5892970001, F. Hoffmann-La Roche Ltd., Mannheim, Germany]) and centrifuged at 13,845 × *g* for 15 min at 4°C. Supernatant samples were collected, and concentration was determined using the BCA Protein Assay Kit. Equal amounts of protein (20 µg) were mixed with 5 × SDS-PAGE sample loading buffer and denatured in boiled H_2_O for 5 min. Protein samples were separated by 12% SDS-PAGE and transferred onto PVDF membranes (IPVHO0010, Merck Ltd., Shanghai, China). Membranes were blocked with Rapid Blocker (G2052-500ML, Servicebio, Wuhan, China) at room temperature (RT) for 30 min, followed by overnight incubation with primary antibodies at 4°C. Afterward, membranes were incubated with HRP-conjugated secondary antibodies at RT for 1 h. Resulting bands were visualized using a multifunctional imaging system (BG-gdsAUTO 710 mini, Baygene Biotech Co., Ltd., China) and semi-quantified with ImageJ (version 1.53i, US National Institutes of Health, USA).

### Chemical crosslinking assay

Cells were treated with EGS crosslinker (the ratio of cell pellet to EGS was 1 mg/ml cells cross linked with 100 µM EGS) in PBS (pH 8.3) for 15 min. Protein samples (30 µg) were extracted and subjected to SDS-PAGE, analyzed by WB using anti-VDAC1 antibody (ab186321, Abcam, Shanghai, China) (17).

### Immunofluorescence assay and stimulated emission depletion imaging

Cells were fixed with 4% paraformaldehyde at RT for 20 min, followed by permeabilization with 0.02% Triton X-100 for 20 min. After blocking with Rapid Blocker for 30 min at RT, cells were incubated overnight with primary antibodies against RSV F protein (sc-101362, Santa Cruz Biotechnology, Dallas, TX, USA, 1:100), VDAC1 (ab186321, Abcam, Shanghai, China, 1:100), and TOM20 (11802-1-AP, Proteintech, Wuhan, China, 1:500) at 4°C. Subsequently, cells were incubated with corresponding secondary antibodies for 1 h at RT. Nuclei were stained with DAPI for 5 min at RT, and after the addition of antifade reagent, coverslips were mounted onto slides. The immunofluorescence (IF) images were collected using an Olympus FLUOVIEW FV1000 confocal laser scanning microscope (Olympus Optical Co., Ltd., Japan), and the mean fluorescence intensity (MFI) was semi-quantified by ImageJ. The stimulated emission depletion (STED) images were collected using a Facility Line Series Super-Resolution Microscope (Light Fine Technology Co., Ltd., China).

### Flow cytometric analysis

#### Apoptosis detection

After treatment, cells were washed twice with ice-cold PBS by centrifugation at 300 × *g* for 5 min at 4°C. Apoptosis was assessed using the Annexin V-FITC/PI Apoptosis Detection Kit (40302ES60, YEASEN, Shanghai, China) following the manufacturer’s instructions and analyzed by flow cytometry (CytoFlex3, Beckman Coulter, Inc., Brea, CA, USA).

#### Intracellular Cl^−^ and Ca^2+^ measurement

DIDS-treated HEp-2 cells were harvested at 48 hpi, incubated with 5 mM MQAE (162558-52-3, MedChemExpress Co., Ltd., Shanghai, China) and 5 μM Fluo-8 AM (MX4505, Shanghai Maokang Co., Shanghai, China) for intracellular Cl^−^ and Ca^2+^ measurements according to the papers published elsewhere (9, 22). The fluorescent signal was examined and analyzed by flow cytometry (CytoFlex3, Beckman Coulter, Inc., Brea, CA, USA).

### Animal experiments

The current study employed a total of 45 specific-pathogen-free (SPF) 8-month-old female BALB/c mice (Huachuang Xinnuo Pharmaceutical Technology Co., Ltd., Jiangsu, China; Experimental Animal Production License no. SCXK [Suzhou] 2020-0009). The mice were categorized into a mock negative control group (*n* = 9), a GZ08-18-infected mice treated with saline group (*n* = 9), a GZ08-18-infected mice treated with 25 mg/kg Ribavirin group (*n* = 9), a GZ08-18-infected mice treated with 12.5 mg/kg DIDS group (*n* = 9), and a GZ08-18-infected mice treated with 25 mg/kg DIDS group (*n* = 9). The mock group received daily intratracheal administration of sterile 0.9% saline for three consecutive days. GZ08-18-infected mice were administered 120 μL of GZ08-18 (equivalent to 1 × 10^8^ TCID_50_) via intratracheal (i.t.) inoculation at 10-12 h intervals for 2 consecutive days, following an established laboratory protocol by our group (19) with minor modification. Two hours after each GZ08-18 administration, mice were intraperitoneally (i.p.) injected with either 0.9% saline, Ribavirin, or DIDS for three consecutive days. The dosages of DIDS and Ribavirin were selected based on previous literature (23, 24). The day of the last GZ08-18 inoculation was set as days post-infection (DPI) 0. The body weight and survival status of each group were daily monitored and recorded until DPI 14.

#### Pulmonary viral load

Bronchoalveolar lavage fluid (BALF) was harvested with DMEM/F12-GlutaMAX containing antibiotics cocktail (20 µg/mL vancomycin, 20 µg/mL ciprofloxacin, 0.05 µg/mL amikacin, 50 µg/mL nystatin, and 1% P/S). The BALF was centrifuged at 13,845 × *g* for 10 min at 4°C, and the supernatant samples were used for TCID_50_ quantification of viral load.

#### Hematoxylin and eosin staining

Lung tissues were fixed in 4% paraformaldehyde, dehydrated, and embedded in paraffin. Sections (4–6 µm) were stained with hematoxylin and eosin (H&E) and imaged using a panoramic slide scanner (Slideview VS200, Olympus Co., Ltd., Japan). Lung injury was assessed based on alveolar structure, septal thickening, and inflammatory cell infiltration.

#### IF assay

Paraffin-embedded lung sections were deparaffinized, rehydrated, antigen-retrieved, blocked with 10% goat serum, and incubated overnight with mouse anti-RSV fusion (F) protein (sc-101362, Santa Cruz Biotechnology, Dallas, TX, USA, diluted to 1:100) at 4°C. Afterward, sections were incubated with fluorescent secondary antibodies for 1 h at 37°C and stained with DAPI for 5 min. Images were captured, and the MFI was semi-quantified by ImageJ.

#### Enzyme-linked immunosorbent assay

Enzyme-linked immunosorbent assay (ELISA) kits were used to measure TNF-α, IFN-γ, and IL-1β expression levels in BALF according to the manufacturer’s instructions. Quantification was performed using the Microplate Reader.

### Data analysis

Raw data were processed, analyzed, and visualized by GraphPad Prism 8.0 (Boston, MA, USA). Normality was assessed with the F-test. For two-group comparisons, Welch’s corrected Student’s *t*-test was applied, and for multiple comparisons, one-way ANOVA followed by Tukey’s post hoc test was used. Data were presented as mean + SD. The *P* values less than and more than 0.05 were considered as statistically significant and non-significant (ns), respectively.

## RESULTS

### DIDS significantly suppresses RSV infection *in vitro*

**Fig 1:**
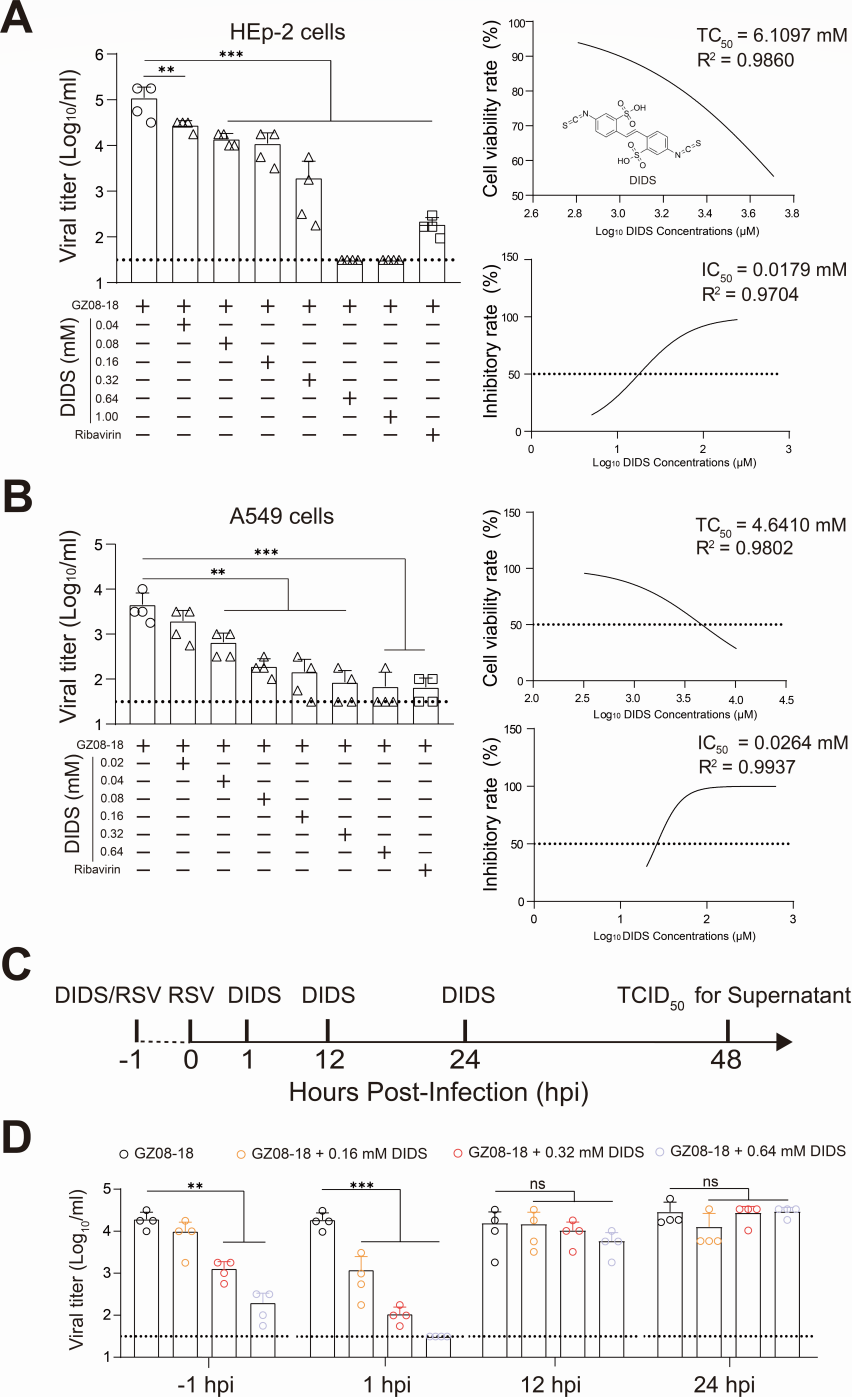
DIDS significantly inhibits RSV infection *in vitro*. (**A and B**) HEp-2 or A549 cells were treated with increasing concentrations of DIDS for 48 h, and TC_50_ was assessed by MTT assay (top right panel). HEp-2 or A549 cells were infected with GZ08-18 at an MOI of 0.1 for 1 h, washed with sterilized PBS, and then exposed to graded concentrations of DIDS in culture medium. Supernatants were harvested at 48 hpi, and viral titer was determined by TCID_50_ assay (left panel). IC_50_ values were calculated from TCID_50_ results (bottom right panel). The chemical structure of DIDS was shown in the TC_50_ data in (**A**). Dashed lines in the left and right panels indicate the detection limit of the TCID_50_ assay and 50% cytotoxicity/inhibition thresholds, respectively. (**C**) TOA assay scheme. GZ08-18 was pre-incubated with 0.16, 0.32, or 0.64 mM DIDS at 37°C for 1 h (DIDS/virus pre-treatment) or added at 1, 12, or 24 hpi. An MOI of GZ08-18 is 0.05. (**D**) Viral titer from groups of GZ08-18, GZ08−18 + 0.16 mM DIDS, GZ08−18 + 0.32 mM DIDS, and GZ08−18 + 0.64 mM DIDS from the indicated time points. The TCID_50_ assay was performed at 48 hpi. The dashed line of (**D**) indicates the detection limit of the TCID_50_ assay. Quantitative data are presented as mean + SD (*n* = 4 per group). Statistical significance is denoted as **P < 0.01 and ***P < 0.001. The ns means non-significance.

To validate the non-cytotoxic concentrations of DIDS for further experiments, we first evaluated the TC_50_ of DIDS in HEp-2 and A549 cells via MTT assay. The TC_50_ values of DIDS were 6.1097 mM and 4.6410 mM against HEp-2 and A549 cells, respectively (Fig. 1A and B, right upper corner panels). Based on TC_50_ results, the anti-RSV activity of DIDS was conducted and evaluated via TCID_50_ assay at 48 hpi. The result showed DIDS significantly suppressed RSV infection in both cell types in a dose-dependent manner. The IC_50_ values of DIDS were 0.0179 mM and 0.0264 mM in HEp-2 and A549 cells against RSV infection, respectively (Fig. 1A and B, left down corner panels), as evidenced by a pronounced reduction in cytopathic effect (CPE) in a dose-dependent manner in Fig. S1A and B. Therefore, the SIs of DIDS anti-RSV in HEp-2 cells and A549 cells were 341.32 and 175.80, respectively. The time-of-addition (TOA) assay was designated as four time points to add DIDS for incubating RSV or HEp-2 cells (Fig. 1C). The results collected via TCID_50_ assay at 48 hpi indicated that DIDS functions mainly as a virucidal agent in the early phase of RSV infection (Fig. 1D), suggesting an anti-RSV mechanism in which DIDS targets a host factor active during the initial phase of infection.

**Fig 2:**
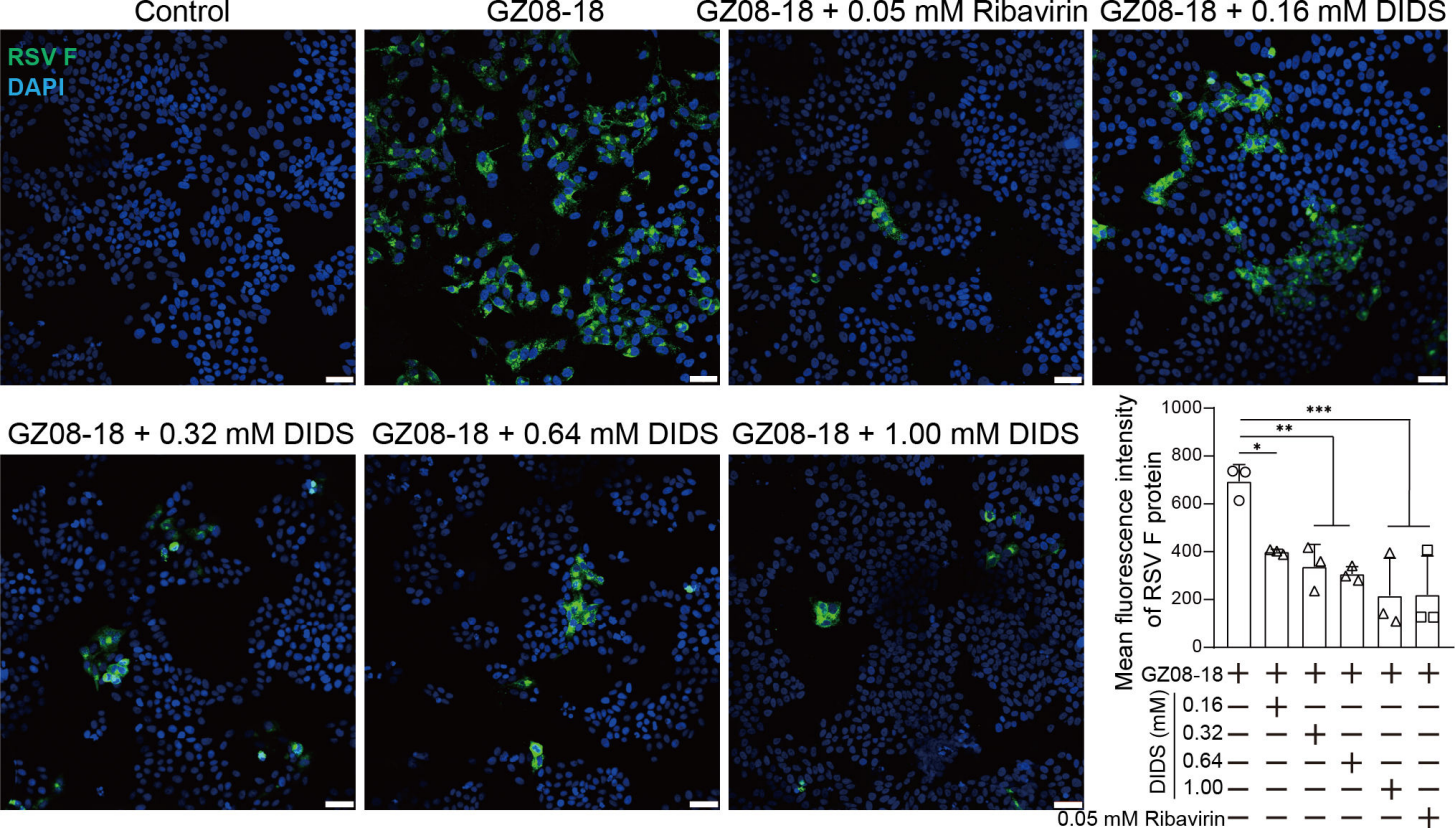
DIDS dose-dependently inhibits RSV F protein expression *in vitro*. HEp-2 cells were infected with GZ08-18 at an MOI of 0.1 for 1 h, washed, and treated with 0.16, 0.32, 0.64, and 1.00 mM DIDS, respectively. At 48 hpi, RSV F protein was detected by IF assay using an anti-F primary antibody (sc-101362, Santa Cruz Biotechnology, Dallas, TX, USA) and Alexa Fluor 488-conjugated secondary antibody (green). Nuclei were counterstained with DAPI (blue). Representative images are shown. MFI of the F protein was semi-quantified using ImageJ (v1.53i, National Institutes of Health, USA) from equal-sized fields of view in IF pictures. Scale bars are 100 μm. Data are presented as mean + SD (*n* = 3 per group). Statistical significance is denoted as *P < 0.05, **P < 0.01, and ***P < 0.001.

To further validate the antiviral activity of DIDS *in vitro*, HEp-2 cells were infected with RSV and treated with various concentrations of DIDS. RSV F protein expression was assessed by IF assay. As shown in Fig. 2, representative IF images were presented. DIDS significantly reduced RSV F protein expression in a dose-dependent manner (Fig. 2, right corner panel).

Taken together, DIDS significantly suppresses RSV infection in both HEp-2 cells and A549 cells.

### DIDS remarkably inhibits lethal RSV infection *in vivo*

**Fig 3:**
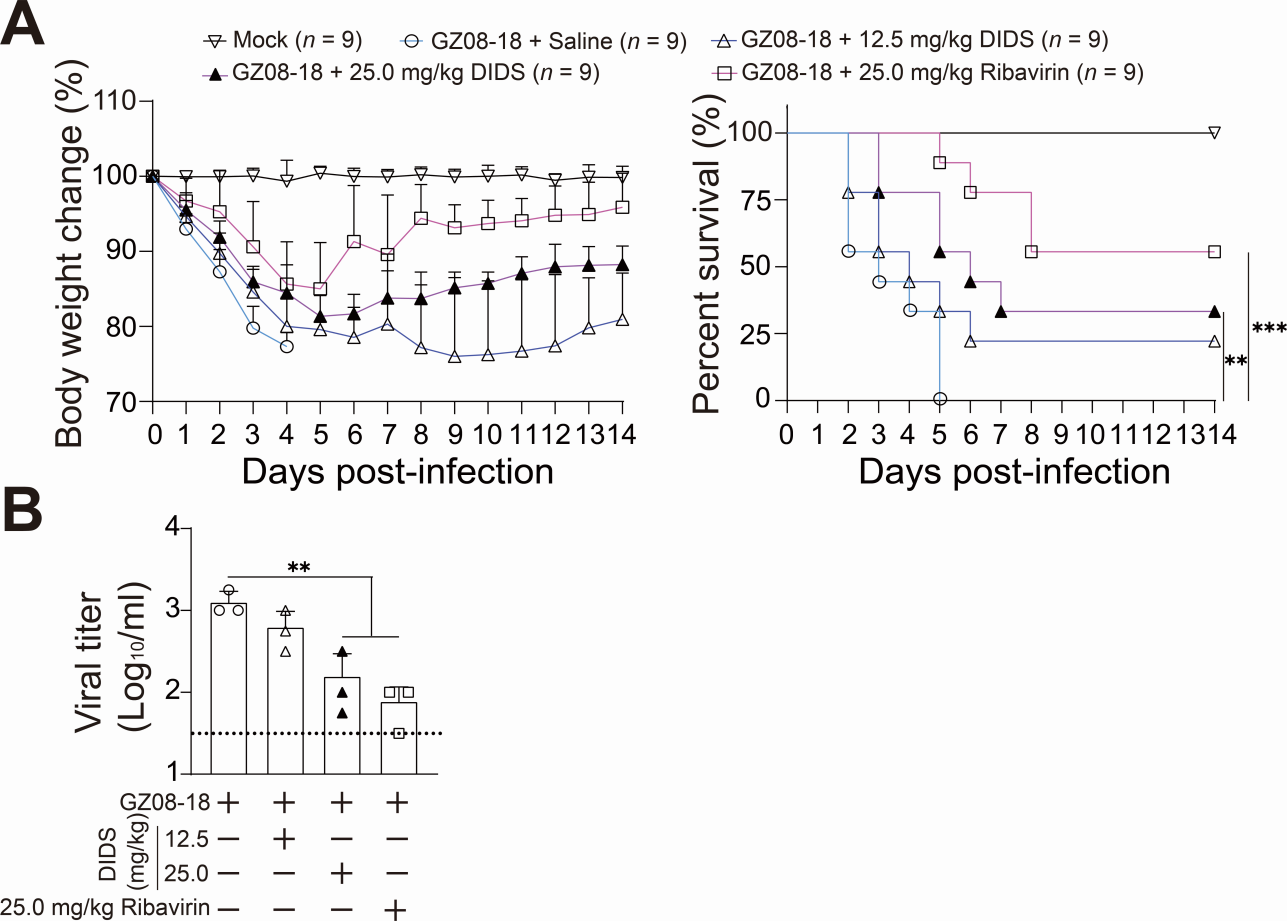
DIDS treatments significantly suppress lethal RSV infection in mice. (**A**) Percentage of mice body weight change and survival rate from groups of Mock, GZ08−18 + Saline (*n* = 9), GZ08−18 + 12.5 mg/kg DIDS (*n* = 9), GZ08−18 + 25.0 mg/kg DIDS (*n* = 9), and GZ08−18 + 25.0 mg/kg Ribavirin (*n* = 9). The mice from GZ08−18 + Saline, GZ08−18 + 12.5 mg/kg DIDS, GZ08−18 + 25.0 mg/kg DIDS, and GZ08−18 + 25.0 mg/kg Ribavirin groups were i.t. inoculated with GZ08-18 (1 × 10^8^ TCID_50_ per administration) three times at 10- to 12-h intervals for two consecutive days. DIDS or Ribavirin treatments were injected i.p. 1 h post-GZ08-18 challenge, following the same three-dose regimen. Mice body weight change (left panel) and survivor rates (right panel) were daily documented and statistically processed by the Log-rank test. **P < 0.01 and ***P < 0.001. (**B**) Viral titer from groups of GZ08−18 + Saline, GZ08−18 + 12.5 mg/kg DIDS, GZ08−18 + 25.0 mg/kg DIDS, and GZ08−18 + 25.0 mg/kg Ribavirin. Mice were sacrificed at 3 dpi. Lung perfusion was conducted, and BALF samples were collected for viral load quantification via TCID_50_ assay. The dashed line indicates the detection limitation of the TCID_50_ assay. Data are presented as mean + SD for (**B**) (*n* = 3 per group). Statistical significance is denoted as **P < 0.01.

The SPF level 8-month-old female BALB/c mice, a well-established lethal mouse model for RSV (GZ08-18) infection (19), were used to evaluate the *in vivo* anti-RSV efficacy of DIDS. The mice were i.t. infected with GZ08-18 (1 × 10^8^ TCID_50_ per administration) three times at 10- to 12-h intervals for two consecutive days. Either 12.5 mg/kg or 25.0 mg/kg DIDS, along with 25.0 mg/kg Ribavirin (positive control), was administered intraperitoneally 1 h after each RSV challenge, following the same three-time regimen for 2 days. All infected groups (except the Control group) exhibited significant weight loss from 0 to 5 dpi. However, the 12.5 mg/kg DIDS, 25.0 mg/kg DIDS, and Ribavirin groups showed weight recovery from 6 to 14 dpi (Fig. 3A, left panel). Strikingly, the GZ08−18 + Saline group mice began dying from 2 dpi, with 100% mortality by 5 dpi (Fig. 3A, right panel). While the 12.5 mg/kg DIDS group showed similar progression, two mice survived the entire experiment. Notably, the 25.0 mg/kg DIDS group demonstrated significant protection against lethal RSV infection, with four survivors, comparable to the Ribavirin group. At 3 dpi, lung tissues and BALF samples were collected for the TCID_50_ assay. The 25.0 mg/kg DIDS treatment significantly reduced viral titers in BALF compared to the GZ08−18 + Saline group, showing similar efficacy to Ribavirin (Fig. 3B).

Taken together, these *in vivo* results demonstrate that DIDS treatment effectively ameliorates disease progression by reducing weight loss and significantly improving survival rates under lethal RSV infection. This protective effect is likely attributable to the substantially reduced viral load observed in BALF samples.

### DIDS treatment alleviates lung pathology, reduces inflammatory responses, and suppresses RSV replication in mice

To further validate the *in vivo* viricidal effects of DIDS, mice were sacrificed at 3 dpi, and lung tissues were collected for histopathological examination and IF analysis.

**Fig 4:**
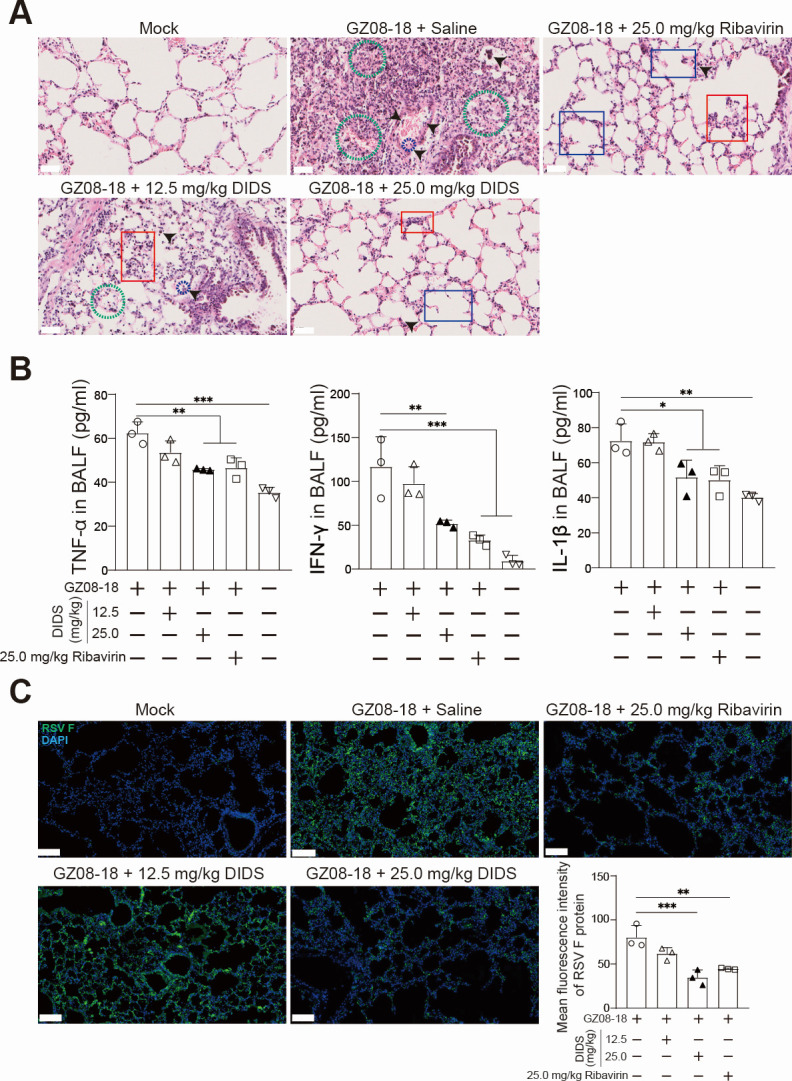
DIDS treatment mitigates pulmonary inflammation and inhibits RSV replication in mice. (**A**) Representative photomicrographs of H&E-stained lung tissue sections. Mice were sacrificed at 3 dpi, and lung tissue sections were prepared and stained using the H&E staining method. Representative photomicrographs showed histopathological changes in 12.5 and 25.0 mg/kg DIDS-treated mice compared to 25.0 mg/kg Ribavirin and RSV-infected controls. Pathological features in (**A**) included alveolar collapse (blue box), thickened alveolar walls (red box), infiltration of inflammatory cells (black bare-head arrows with or without blue dashed circle), and erythrocyte extravasation into alveolar spaces (green dashed circle). Scale bars are 100 μm. (**B**) Mice were sacrificed at 3 dpi. Lung perfusion was conducted, and BALF samples were collected. The concentrations (pg/mL) of TNF-α, IFN-γ, and IL-1β in BALF samples were quantified by ELISA assay. (**C**) Representative IF photomicrographs of lung tissue sections. IF staining showed RSV F protein expression (green, sc-101362, Santa Cruz Biotechnology, Dallas, TX, USA) in lung tissues following DIDS or Ribavirin administration, with nuclei counterstained using DAPI (blue). Scale bars are 100 μm. MFI of the F protein was semi-quantified by ImageJ (v1.53i, National Institutes of Health, USA) from equal-sized fields of view in images. Quantitative data are presented as mean + SD (*n* = 3 per group). Statistical significance is indicated as *P < 0.05, **P < 0.01, and ***P < 0.001.

As shown in Fig. 4A, histopathological examination by H&E staining demonstrated RSV infection induced extensive pulmonary impairments, including alveolar collapse, thickened alveolar walls, infiltration of inflammatory cells, and erythrocyte extravasation into alveolar spaces. DIDS and Ribavirin treatments markedly alleviated these pathological changes, reducing both alveolar structural damage and inflammatory infiltration. Treating with 25.0 mg/kg DIDS mitigated epithelial cell desquamation, necrosis, edema, and inflammatory infiltration of the tracheal submucosa, compared with the 25.0 mg/kg Ribavirin group and Mock group. Consistent with these histopathological observations, expression levels of pro-inflammatory cytokines, including TNF-α, IFN-γ, and IL-1β in BALF, were significantly decreased in the DIDS-treated groups (Fig. 4B). Moreover, the IF assay demonstrated a substantial reduction in RSV F protein expression in lung tissues post-DIDS administration (Fig. 4C), compared with RSV controls. Collectively, these results further verify that DIDS exerts potent *in vivo* antiviral activity against lethal RSV infection.

### VDAC1 facilitates RSV infection in HEp-2 cells

**Fig 5:**
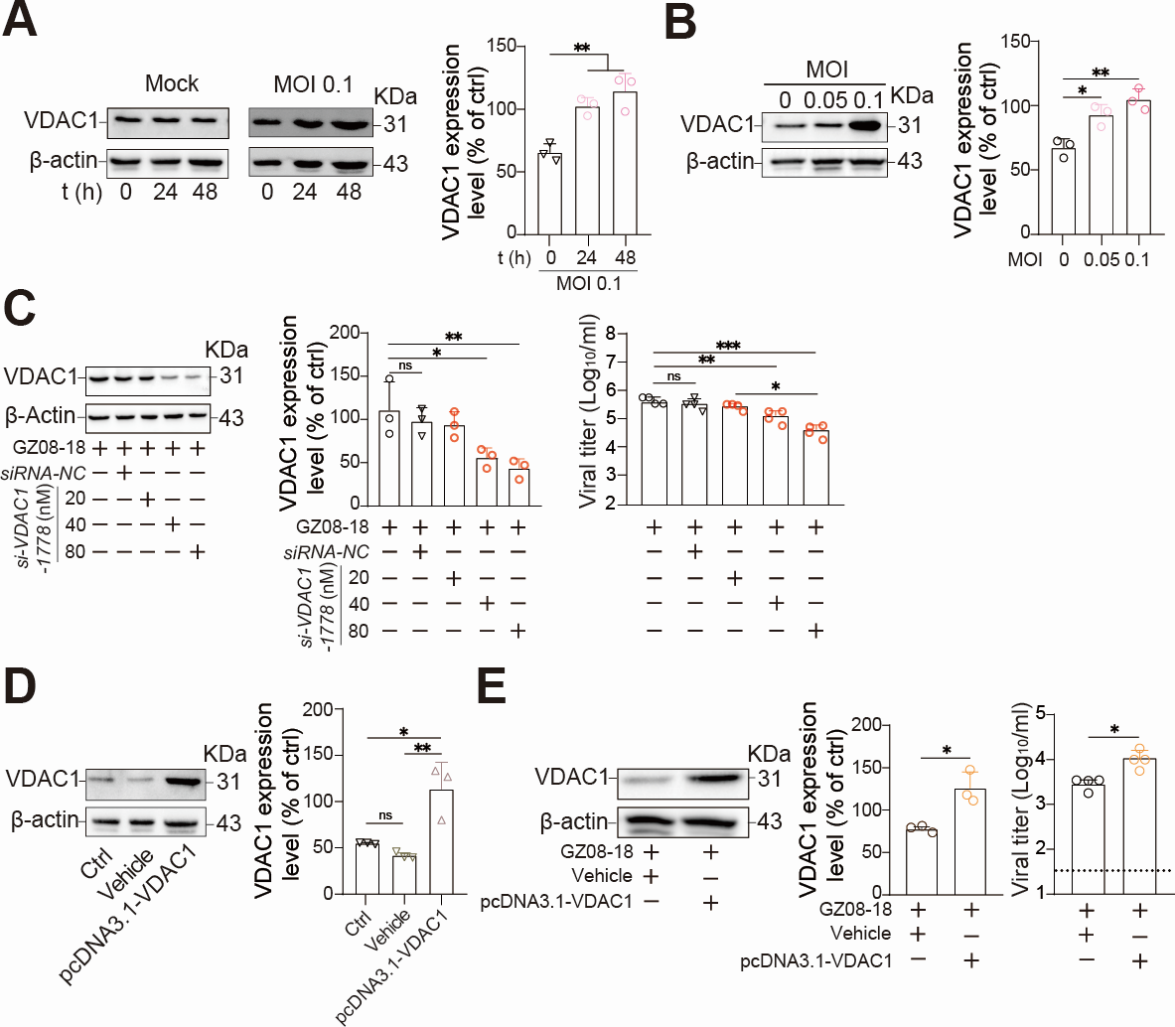
VDAC1 facilitates RSV infection in HEp-2 cells. (**A**) HEp-2 cells were infected with GZ08-18 at an MOI of 0.1. VDAC1 expression was assessed at 0, 24, and 48 hpi via WB assay (left panel). Semi-quantification of VDAC1 expression level was performed using ImageJ (v1.53i, National Institutes of Health, USA) (right panel). (**B**) HEp-2 cells were infected with GZ08-18 at 0, 0.05, and 0.1 MOI for 48 h, respectively. VDAC1 expression in cell lysates was analyzed by WB assay (left panel). The expression level of VDAC1 was semi-quantified by ImageJ (right panel). (**C**) HEp-2 cells were transfected with 20, 40, and 80 nM *si-VDAC1-1778* or a negative control *siRNA-NC* (80 nM). At 24 h post-transfection, cells were infected with GZ08-18 at an MOI of 0.1 for 1 h. At 48 hpi, VDAC1 knockdown was checked by WB assay (left panel). The expression level of VDAC1 was semi-quantified by ImageJ (middle panel). GZ08-18 replication was assessed by the TCID_50_ assay (right panel). (**D**) Fixed numbered HEp-2 cells were pre-seeded in a 12-well plate and transfected with 2.5 µg pcDNA3.1-VDAC1 per well. At 48 h post-transfection, VDAC1 overexpression was determined by WB assay (left panel). The expression level of VDAC1 was semi-quantified by ImageJ (right panel). Untreated (Ctrl) and empty plasmid (Vehicle) groups served as controls. (**E**) HEp-2 cells were infected with GZ08-18 at an MOI of 0.1 for 48 h. After validating VDAC1 overexpression by WB assay (left panel) and ImageJ for semi-quantification of VDAC1 expression levels (middle panel), GA08-18 replication was quantified by the TCID_50_ assay (right panel). β-actin served as the loading control. Quantitative data are presented as mean + SD (*n* = 3 or 4 per group). Statistical significance was determined as *P < 0.05, **P < 0.01, and ***P < 0.001. The ns means non-significance.

DIDS was reported as the inhibitor not only for VDAC1 (25) but also for RAD51 (26). Consequently, we sought to investigate whether DIDS treatment could alter RAD51 expression in HEp-2 cells following GZ08-18 infection. WB analysis, as depicted in Fig. S2, revealed no change in RAD51 expression. Furthermore, RAD51 has been rarely associated with respiratory viruses in published literature. Based on these findings, we hypothesize that RSV infection leads to fluctuations in VDAC1 expression, which subsequently promotes viral replication. To test it, we measured VDAC1 expression levels in 0.1 MOI GZ08018-infected HEp-2 cells at 0, 24, and 48 hpi via WB assay (Fig. 5A). We also infected HEp-2 cells with 0, 0.05, and 0.1 MOI GZ08-18 and examined their VDAC1 expression levels at 48 hpi via WB assay (Fig. 5B). The results demonstrated a significant and time-/dose-dependent increase in VDAC1 expression upon GZ08-18 infection (Fig. 5A and B). To further investigate the role of VDAC1 in RSV infection, we screened *si-VDAC1-1778* out from four *si-VDAC1*s to silence the decoding of *VDAC1* (Fig. S3A and B). Transfection of *si-VDAC1-1778* into HEp-2 cells significantly suppressed *VDAC1* decoding and inhibited RSV infection in a dose-dependent manner, while the performance of the *siRNA-NC* group matched that of the GZ08-18 group, confirming no non-specific effects of the siRNA treatment (Fig. 5C). Subsequently, pcDNA3.1-VDAC1 was introduced into HEp-2 cells, and the statistically overexpression of VDAC1 was confirmed in pcDNA3.1-VDAC1-transfected cells (Fig. 5D). In contrast with VDAC1 silencing results (Fig. 5C), but as expected, the viral infection was significantly increased in pcDNA3.1-VDAC1-transfected cells (Fig. 5E). These results highly suggest VDAC1 facilitates RSV infection in HEp-2 cells.

### DIDS suppresses RSV-induced VDAC1 oligomerization in the mitochondrial membrane and apoptosis to limit viral replication

**Fig 6:**
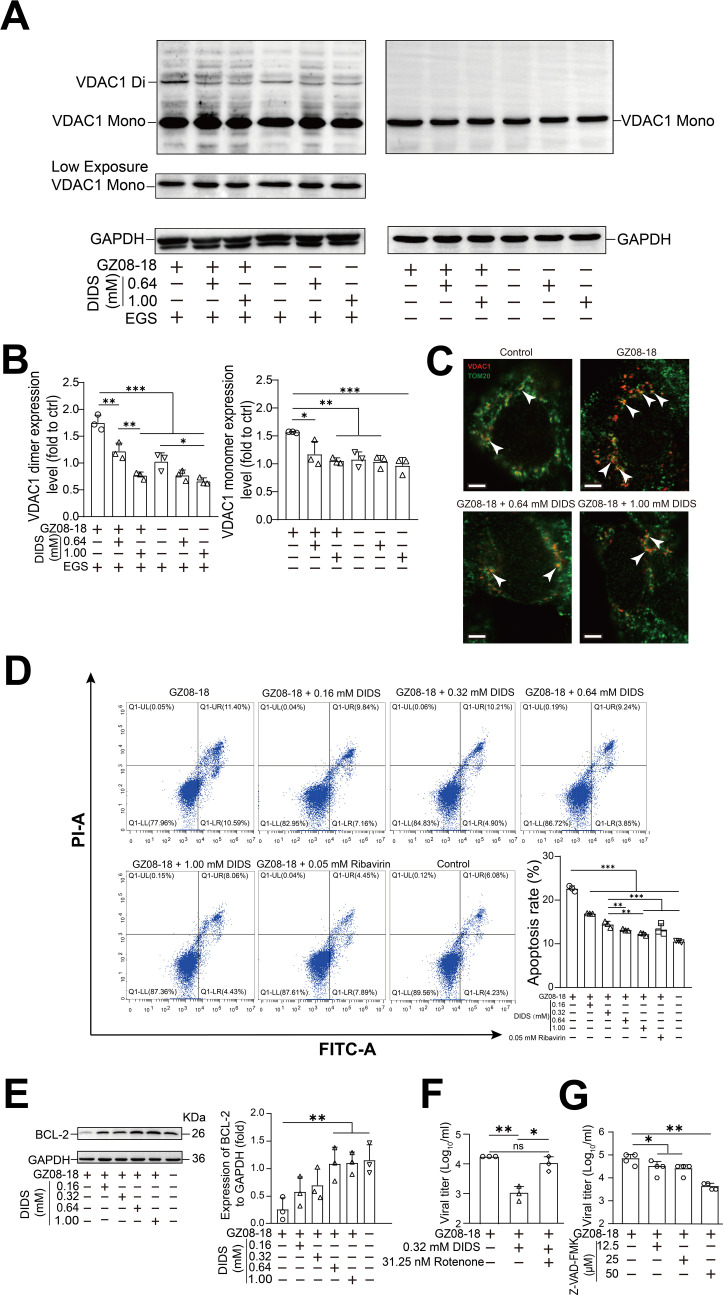
DIDS inhibits RSV-induced mitochondrial VDAC1 oligomerization and apoptosis. (**A**) WB results of the crosslinking assay. HEp-2 cells were infected with GZ08-18 at an MOI of 0.1 for 1 h, after which the inoculum was removed and replaced with medium containing 0.64 and 1.00 mM of DIDS. At 48 hpi, cell lysates were collected and lysed in pre-cooled RIPA buffer for extraction of general protein. For oligomerization analysis, general protein samples were crosslinked with EGS in PBS (pH = 8.3) for 15 min prior to immunoblotting for VDAC1 (left panel). Meantime, parallel general protein samples without EGS served as controls (right panel). The positions of VDAC1 monomers and multimers are indicated as VDAC1 monomer (VDAC1 Mono) and VDAC1 dimer (VDAC1 Di), respectively. The low exposure of VDAC1 was presented alone, whereas the over-exposed VDAC1 was shown to demonstrate the VDAC1 oligomerization with the treatment of EGS (left panel). (**B**) Expression levels of dimers and monomers of VDAC1 were semi-quantified by ImageJ (v1.53i, National Institutes of Health, USA). (**C**) HEp-2 cells were infected with GZ08-18 at an MOI of 0.1 for 1 h, washed, and treated with 0.64 and 1.00 mM DIDS, respectively. At 48 hpi, the IF assay was conducted by primary antibodies against VDAC1 (ab186321, Abcam, Shanghai, China, 1:100) and TOM20 (11802-1-AP, Proteintech, Wuhan, China, 1:500), then by the Alexa Fluor 488-conjugated secondary antibody (green) and the Alexa Fluor 594-conjugated secondary antibody (red), respectively. Images were captured by STED microscopy, and representative images are shown. White barehead arrows point to regions of VDAC1-TOM20 overlap (yellow). Scale bars are 5 μm. (**D**) Apoptosis rate from groups of GZ08-18, GZ08−18 + 0.16 mM DIDS, GZ08−18 + 0.32 mM DIDS, GZ08−18 + 0.64 mM DIDS, GZ08−18 + 1.00 mM DIDS, GZ08−18 + 0.05 mM Ribavirin, and Control. HEp-2 cells were infected with GZ08-18 at an MOI of 0.1 for 1 h, after which the inoculum was removed and replaced with medium containing 0.16, 0.32, 0.64, and 1.00 mM of DIDS, and 0.05 mM Ribavirin as a positive control. A group of blank cells without treatments of GZ08-18, DIDS, and Ribavirin served as a control. Apoptosis rate was assessed from grouped cells by flow cytometry (CytoFlex3, Beckman Coulter, Inc., Brea, CA, USA) with the Annexin V-FITC/PI Apoptosis Detection Kit (40302ES60, YEASEN, Shanghai, China) at 48 hpi. (**E**) WB result of BCL-2 protein levels. HEp-2 cells were infected with GZ08-18 at an MOI of 0.1 for 1 h, after which the inoculum was removed and replaced with medium containing 0.16, 0.32, 0.64, and 1.00 mM of DIDS. BCL-2 protein levels were assessed by WB assay at 48 hpi and semi-quantified by ImageJ with the GAPDH as loading control. (**F**) Viral titer from groups of GZ08-18, GZ08−18 + 0.32 mM DIDS, and GZ08−18 + 0.32 mM DIDS + 31.25 nM Rotenone. HEp-2 cells were infected with GZ08-18 at an MOI of 0.1 for 1 h, after which the inoculum was removed and replaced with medium for the GZ08-18 group, or with medium containing 0.32 mM DIDS for the GZ08−18 + 0.32 mM DIDS group. As for the GZ08−18 + 0.32 mM DIDS + 31.25 nM Rotenone group, the medium was removed again and replaced with medium containing 31.25 nM Rotenone at 12 hpi. Viral titers were determined by TCID_50_ assay at 48 hpi. (**G**) HEp-2 cells were infected with GZ08-18 at an MOI of 0.1 for 1 h, after which the inoculum was removed and replaced with medium containing 12.5, 25.0, and 50.0 μM Z-VAD-FMK, respectively, and viral titers were measured by the TCID_50_ assay at 48 hpi. Quantitative data are presented as mean + SD (*n* = 3 or 4 per group). Statistical significance was determined as *P < 0.05, **P < 0.01, and ***P < 0.001. The ns means non-significance.

VDAC1 oligomerizes in the mitochondrial outer membrane under apoptotic stress, forming large pores that regulate intrinsic apoptosis (27). DIDS was reported to inhibit VDAC1 oligomerization, therefore suppressing apoptosis (17). We hypothesized that RSV harnesses VDAC1 oligomerization to facilitate its replication. Thus, we employed a cross-linking assay coupled with immunoblotting to quantify VDAC1 oligomerization levels in the presence and absence of DIDS. Notably, the VDAC1 dimer—an indicator of oligomerization—was significantly more abundant in the RSV-infected group compared to the group treated with EGS alone. Furthermore, the addition of 0.64 or 1.00 mM DIDS resulted in a marked reduction in VDAC1 dimer formation with a gradually decreased pattern in VDAC1 dimer, relative to RSV-infected controls (left panel of Fig. 6A and B). As expected, the EGS-free treatment groups showed no significant effect on VDAC1 oligomerization (right panel of Fig. 6A and B). We further employed super-resolution STED microscopy to directly visualize the spatial distribution and clustering of VDAC1 and TOM20 on mitochondrial surfaces (Fig. 6C). Visual inspection of the STED images revealed that RSV infection notably increased the co-localization of VDAC1 and TOM20 on mitochondrial membranes, reflecting enhanced association of VDAC1 with this subcellular compartment during infection. In contrast, treatment with 0.64 or 1.00 mM DIDS resulted in a marked reduction in the co-localization of VDAC1 and TOM20 compared to Control or RSV-infected cells (Fig. 6C). Given that elevated VDAC1 oligomerization is associated with cellular apoptosis (17), these findings (Fig. 6A through C) highly suggested DIDS mitigates RSV-induced apoptosis, thereby attenuating viral infection. To validate it, the result of flow cytometry analysis confirmed DIDS treatment significantly suppressed RSV-triggered apoptosis in HEp-2 cells (Fig. 6D). Consistent with this, immunoblotting revealed BCL-2—an anti-mitochondrial apoptotic protein—was expressed at significantly higher levels in GZ08-18-infected cells treated with 0.16, 0.32, 0.64, and 1.00 mM DIDS, in a dose-dependent manner, relative to RSV-infected controls (Fig. 6E). Next, we sought to directly assess whether apoptosis supported RSV replication. HEp-2 cells were treated with or without 0.32 mM DIDS at 12 hpi, followed by medium replacement and addition of the pro-intrinsic apoptotic agent Rotenone (28, 29). At 48 hpi, viral titers measured via TCID_50_ assay demonstrated Rotenone restored RSV replication, abrogating the antiviral effect of DIDS (Fig. 6F). Conversely, post-infection treatment with the pan-caspase inhibitor Z-VAD-FMK (30) significantly reduced RSV titers (Fig. 6G). Taken together, these results demonstrate that RSV promotes mitochondrial VDAC1 oligomerization-driven apoptosis to facilitate its replication, and DIDS impairs this proviral pathway by reducing VDAC1 oligomerization and then inhibiting apoptosis.

### Exogenous Cl^−^ supplementation enhances RSV replication

**Fig 7:**
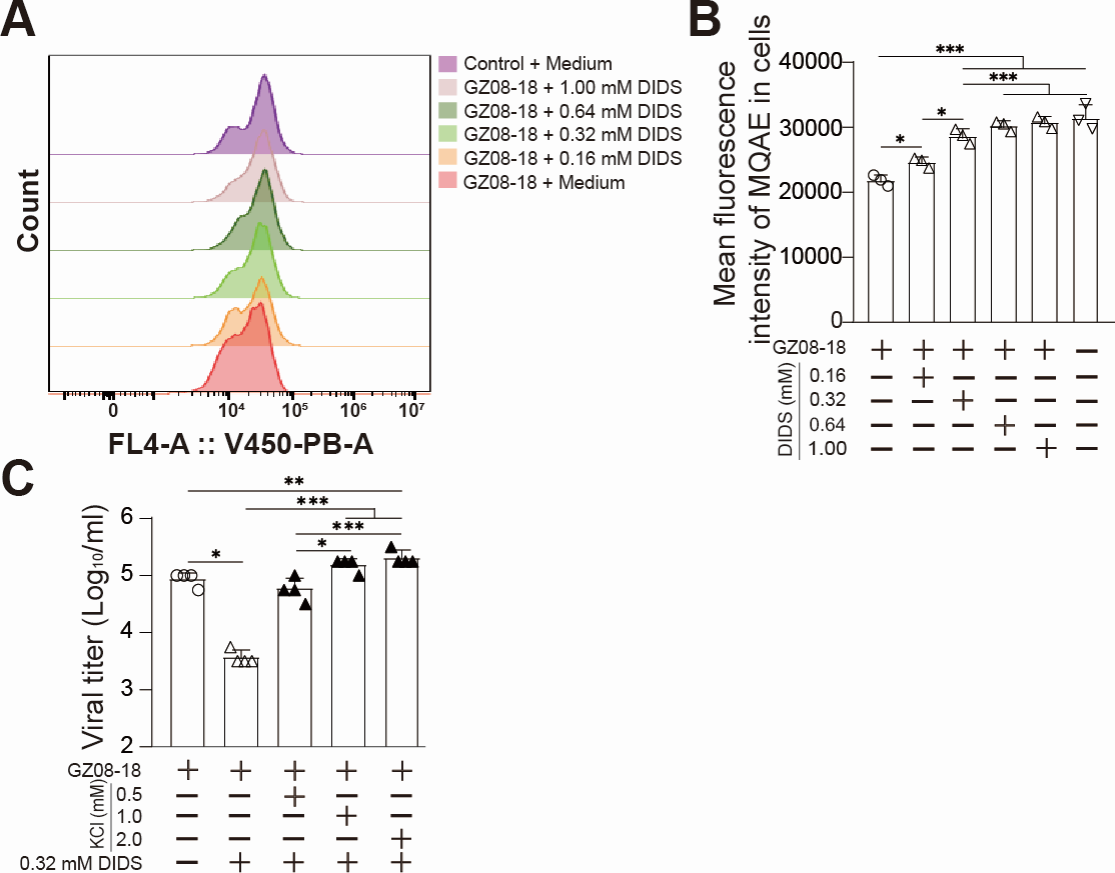
Exogenous Cl^−^ supplementation enhances RSV replication. (**A**) Flow diagram of intracellular Cl^−^ fluorescence intensity from groups of Control + Medium, GZ08−18 + 1.00 mM DIDS, GZ08−18 + 0.64 mM DIDS, GZ08−18 + 0.32 mM DIDS, GZ08−18 + 0.16 mM DIDS, and GZ08−18 + Medium. HEp-2 cells were infected with GZ08-18 at an MOI of 0.1 for 1 h. The supernatant was removed and replaced with medium containing 1.00, 0.64, 0.32, and 0.16 mM DIDS. At 48 hpi, cell supernatants were collected, and intracellular Cl^−^ levels were measured by flow cytometry (CytoFlex3, Beckman Coulter, Inc., Brea, CA, USA) using 5 mM MQAE (162558-52-3, MedChemExpress Co., Ltd., Shanghai, China). (**B**) Quantification of MFI of MQAE in cells in groups of GZ08−18 + Medium, GZ08−18 + 0.16 mM DIDS, GZ08−18 + 0.32 mM DIDS, GZ08−18 + 0.64 mM DIDS, GZ08−18 + 1.00 mM DIDS, and Control + Medium. MQAE is a non-ratiometric, Cl^−^-quenched fluorescent indicator used to quantify intracellular Cl^−^ concentrations. Its fluorescence intensity increases with decreasing intracellular Cl^−^ levels. (**C**) Viral titer from groups of GZ08−18 + Medium, GZ08−18 + 0.32 mM DIDS, GZ08−18 + 0.5 mM KCl + 0.32 mM DIDS, GZ08−18 + 1.0 mM KCl + 0.32 mM DIDS, and GZ08−18 + 2.0 mM KCl + 0.32 mM DIDS. HEp-2 cells were infected with GZ08-18 at an MOI of 0.1 for 1 h. Then the culture medium was replaced with fresh media containing 0.5, 1.0, and 2.0 mM KCl plus 0.32 mM DIDS, or solo 0.32 mM DIDS, respectively. The TCID_50_ assay was performed to detect RSV titer in supernatant samples at 48 hpi. Quantitative data are presented as mean + SD (*n* = 3 or 4 per group). Statistical significance was determined as *P < 0.05, **P < 0.01, and ***P < 0.001.

VDAC1, a key anion channel in the mitochondrial outer membrane, regulates ion flow between the mitochondria and cytosol in its oligomeric state (31). We have verified RSV infection-induced mitochondrial VDAC1 oligomerization (Fig. 6A through C), thereby altering host intracellular ion composition and promoting viral replication, which was theoretically worked. To explore it, flow cytometric analysis was performed to measure intracellular Cl^-^ and Ca^2+^ levels post-RSV infection and DIDS administration. A dose-dependent DIDS in decreasing intracellular Cl^−^ was observed (Fig. 7A and B), while no significant changes in intracellular Ca^2+^ levels were detected (Fig. S4A and B). When the 0.5, 1.0, and 2.0 mM KCl plus 0.32 mM DIDS were added into GZ08-18-infected cells, viral titers of KCl-treated groups at 48 hpi were dramatically increased in a dose-dependent manner, in contrast with the solo DIDS-treated group. The viral titer of 2.0 mM KCl plus 0.32 mM DIDS group was extremely higher than the RSV control group and the DIDS group (Fig. 7C). Likewise, as shown in Fig. S4C, the exogenous supplementation with CaCl_2_ plus DIDS, promoted RSV propagation. Notably, no statistically significant difference was observed between the CaCl_2_ plus DIDS treatment group and the virus control group. Following EGTA addition, the 0.1 mM CaCl_2_ plus DIDS/EGTA group exhibited a statistically significant increase in viral titer relative to the virus control group, whereas no significant difference was detected when compared with the 0.1 mM CaCl_2_ plus DIDS (EGTA-free) group. Collectively, these results suggest that supplementing with exogenous Cl^−^, rather than Ca²^+^, plays a critical role in DIDS-modulated RSV pathogenesis. These data strongly implicate the VDAC1-regulated Cl^−^ channel as a promising target for antiviral intervention.

## DISCUSSION

**Fig 8:**
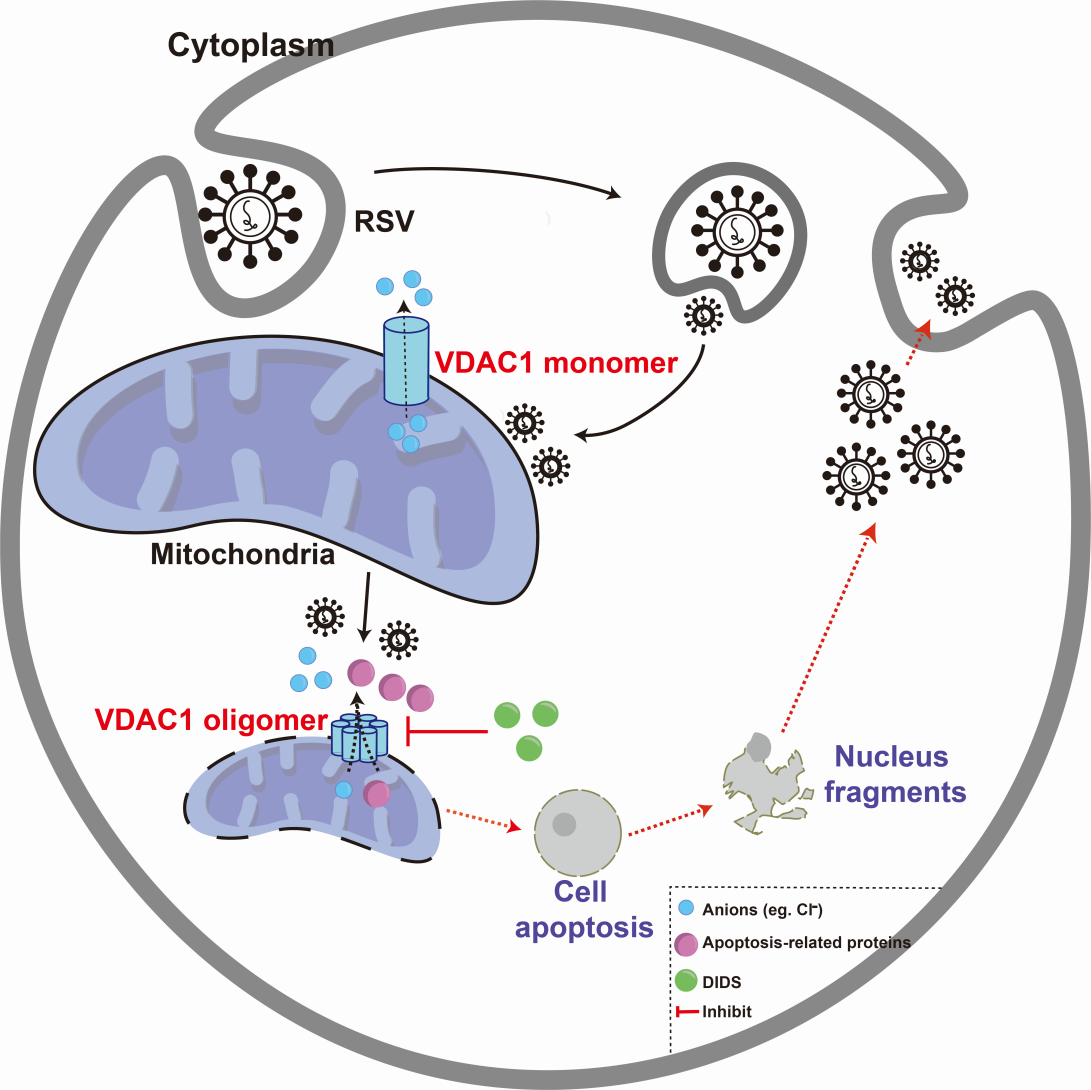
DIDS modulates VDAC1 oligomerization to suppress mitochondrial intrinsic apoptosis and attenuate RSV infection. DIDS modulates VDAC1 oligomerization to suppress cellular mitochondrial intrinsic apoptosis and maintain chloride ion homeostasis, ultimately inhibiting viral infection.

The regulation of host cell ion channel activity by viruses has emerged as a critical element for virus-host interactions (4). Viruses harness ion channels to facilitate the entry and exit of viral progeny, thereby maintaining a cellular environment conducive to viral persistence (32). Cumulative evidence indicates that pharmacological targeting of ion channels can effectively disrupt the viral life cycle (33). In this study, we demonstrate that the DIDS, a well-established chloride channel blocker and an inhibitor of VDAC1, significantly inhibits VDAC1 oligomerization that is triggered by RSV infection. This inhibition reduces mitochondrial intrinsic apoptosis, restores chloride ion homeostasis, and ultimately attenuates viral infection (Fig. 8).

Pearson et al. previously reported that DIDS exhibited an inhibitory effect on RSV infection *in vitro*. Specifically, they demonstrated that 0.02 mM and 0.04 mM DIDS achieved approximately 60% and 80% inhibition rates against RSV infection in A549 cells, accompanied by a marked suppression of viral G protein expression. Interestingly, the TOA assay performed by Pearson et al. indicated NPPB, another established chloride channel blocker, exerted protective effects against RSV infection during the 0–9 hpi window (18). Coincidentally, the current study also confirmed that DIDS significantly reduced RSV infection *in vitro*, as evidenced by a notable decrease in viral titer, CPE, and expression of viral F protein. We observed that 0.02 mM and 0.04 mM DIDS yielded inhibition rates of approximately 56.4% and 85.5% against RSV infection in A549 cells, mirroring the previous observations. Furthermore, our TOA data underscored an effective virucidal effect of DIDS at −1 and 1 hpi, but no effect at 12 and 24 hpi. This difference in effective scavenging time points may be attributable to distinct cellular or viral targets engaged by DIDS and NPPB during RSV infection. In a separate context, Cardin et al. reported DIDS inhibits HIV-1 infection in CD4^+^ T cells with an IC_50_ of 0.03 mM (34). Analogously, we determined IC_50_ values of 0.0179 mM and 0.0264 mM for DIDS against RSV in HEp-2 and A549 cells, respectively. Moreover, the SI of 341.32 and 175.80 derived from the DIDS anti-RSV assays in HEp-2 and A549 cells, respectively, suggest a favorable druggable potential for DIDS as an antagonist against RSV infection.

*In vivo* assessments of DIDS’s antiviral efficacy have been reported in several studies for various viruses. For instance, studies involving chickens infected with infectious bronchitis virus (IBV) demonstrated that oral gavage administration of 0.15 mM and 0.45 mM DIDS resulted in significantly increased survival rates of 33.3% and 83.3%, respectively, compared to a 0% survival rate observed in the untreated IBV control group. Histopathological examination of tracheal sections stained with H&E indicated that DIDS notably alleviated tracheal lesions, characterized by reduced inflammatory cell infiltration (35). Furthermore, in models of spring viremia of carp virus (SVCV) infection—which is associated with pyroptosis and NLRP3 inflammasome activation—treatment with 0.02 mM DIDS significantly increased the survival rate of zebrafish to approximately 80%, compared to only 20% survival in the SVCV-infected group (36). Consistent with these findings, our mouse model experiments demonstrated that DIDS at doses of 12.5 mg/kg and 25 mg/kg (equivalent to approximately 7 mM and 14 mM, respectively) exhibited strong anti-RSV effects, including alleviation of weight loss, substantial improvement in survival rates, and reduction of histopathological changes and inflammation in pulmonary tissue compared to the RSV control group.

To elucidate whether DIDS-mediated blockade of viral infection involved the disruption of RAD51 in addition to VDAC1, we performed a preliminary WB analysis to evaluate RAD51 expression level. The assay revealed consistent expression levels across the blank control, RSV control, and RSV plus DIDS treatment groups. This lack of effect on RAD51 leads us to hypothesize that VDAC1 is the principal target through which DIDS modulates RSV infection.

VDAC1, predominantly localized to the mitochondrial outer membrane, is well established to undergo oligomerization in response to apoptotic stimuli, thereby forming large pores that govern the intrinsic apoptotic pathway (37). While VDAC1 had been implicated in numerous viral infections, the precise mechanisms underpinning its involvement have remained elusive, largely confined to several hypothetical models (32). For instance, Zamarin et al. demonstrated that the influenza virus PB1-F2 protein interacts with VDAC1 and adenine nucleotide translocase 3 (ANT3) to facilitate the opening of the mitochondrial permeability transition pore, leading to the release of apoptogenic factors and subsequent apoptosis (38). Similarly, HIV-1 Vpr has been shown to interact with VDAC1 to induce T-cell apoptosis, a process that aids viral replication and immune evasion (39). Motivated by these precedents, we hypothesized that VDAC1 played a crucial role in RSV infection. Our experimental data strongly supported this premise that the RSV infection significantly upregulated VDAC1 expression in a time- and virus dose-dependent manner. Furthermore, both gain-of-function and loss-of-function studies confirmed that VDAC1 overexpression significantly enhanced viral replication in HEp-2 cells, whereas the silencing of VDAC1 dramatically inhibited infection. Coupled with the early-phase virucidal effect observed for DIDS in the TOA assay, these findings provide clear experimental evidence establishing VDAC1 as a potentially key player in RSV pathogenesis.

To further delineate this process, functional intervention experiments were conducted via DIDS in HEp-2 cells. Our results showed RSV infection enhanced VDAC1 oligomerization, which was associated with increased apoptosis (40). Supported by both EGS crosslinking WB assays and STED microscopy data, treatment with DIDS effectively blocked RSV-induced VDAC1 oligomerization. STED images revealed that RSV infection increased VDAC1/TOM20 co-localization on mitochondrial membranes, whereas DIDS treatment markedly reduced this overlap, thereby disrupting the VDAC1 oligomerization on mitochondrial membranes. Concomitantly, DIDS diminished RSV-induced apoptosis, collectively indicating that VDAC1 oligomerization plays a critical role in promoting viral replication. Additional findings, such as pro-mitochondrial apoptotic agent Rotenone, significantly exacerbated RSV infection, whereas the anti-apoptotic agent Z-VAD-FMK notably inhibited RSV infection, further verifying these observations. These results collectively suggested that VDAC1-induced apoptosis is a key mechanism driving viral replication. As an anion channel, VDAC1 not only regulates the release of apoptosis-related proteins from the mitochondrial intermembrane space but also plays a role in maintaining intracellular ion homeostasis (31). It was hypothesized that RSV infection-induced VDAC1 oligomerization could alter ion flux through the channel. Experimental results revealed RSV infection increased VDAC1 expression, shifting the equilibrium toward the oligomeric state, which promoted the release of Cl^−^ and Ca^2+^ into the cytoplasm, leading to alterations in mitochondrial membrane potential and triggering apoptosis. Interestingly, after treatment with DIDS, both Cl^−^ and Ca^2+^ levels in the cytoplasm were significantly reduced, with only Cl^−^ concentrations showing a significant dose-dependent decrease. Furthermore, the addition of KCl plus DIDS treatment in the context of viral infection resulted in a marked recovery of viral replication, suggesting exogenous Cl^−^ supplementation could overturn the decrease of Cl^−^ flux caused by DIDS and promote RSV replication.

Several limitations existed in the present study. A primary constraint is the reliance on DIDS as a functional inhibitor. Future work must incorporate highly specific VDAC1 blockers, such as VBIT-3 or VBIT-4, to unequivocally confirm VDAC1’s essentiality and rule out possible contributions from VDAC2 (36) or VDAC3 during RSV infection. Besides, DIDS exhibited favorable anti-RSV activity *in vitro* but less robust performance *in vivo*. While this discrepancy may stem from inherent differences between *in vitro* and *in vivo* environments, it highlights the need to advance DIDS as a lead compound—specifically via AI-driven conformational modifications—to enhance its *in vivo* anti-RSV activity. Furthermore, while MQAE-based flow cytometry supported Cl^−^ flux observations, utilizing whole-cell patch clamp techniques is recommended to acquire the superior, single-channel kinetic resolution necessary for detailed ion channel characterization. Finally, direct *in vivo* data showing VDAC1 oligomerization dynamics correlating with apoptosis in the current context were absent. Future research should focus on rectifying these issues.

Overall, our data confirmed mitochondrial VDAC1 oligomerization was critical for RSV-induced intrinsic apoptosis and viral pathogenesis, with the inhibitor DIDS effectively disrupting this process to reduce viral load *in vitro* and *in vivo*. By preventing VDAC1 oligomerization and subsequent mitochondrial pathway apoptosis, DIDS intervention offered critical mechanistic insight into VDAC1 function during infection. Targeting mitochondrial anion channels like VDAC1 thus represents a promising strategy for developing innovative anti-RSV therapeutics.

## Data Availability

All data generated and analyzed during this study are described in the article. The intermediate and supporting data will be available from the corresponding authors (Prof. Yu-Si Luo & Prof. Ke Zhang) upon reasonable request.

## SUPPLEMENTAL MATERIAL

Fig. S1 jvi.02200-25-s0001.docx DIDS suppresses RSV-induced CPE in HEp-2 and A549 cells.

Fig. S2 jvi.02200-25-s0002.docx DIDS does not cause RAD51 expression change upon RSV infection.

Fig. S3 jvi.02200-25-s0003.docx Verification of siRNA-mediated VDAC1 knockdown.

Fig. S4 jvi.02200-25-s0004.docx DIDS does not cause intracellular Ca^2+^ influx upon RSV infection.

Table S1 jvi.02200-25-s0005.docx siRNA sequences of this study.

ASM does not own the copyrights to Supplemental Material that may be linked to, or accessed through, an article. The authors have granted ASM a non-exclusive, world-wide license to publish the Supplemental Material files. Please contact the corresponding author directly for reuse.
