# Supplementary material for: DIDS modulates VDAC1 oligomerization to suppress intrinsic apoptosis and attenuates in vitro and in vivo RSV infection
Source: J Virol. 2026 Feb 11;100(3):e02200-25. doi: 10.1128/jvi.02200-25 (PMC13011466; doi:10.1128/jvi.02200-25)
Supplement: Fig. S1 — DIDS suppresses RSV-induced CPE in HEp-2 and A549 cells. [file jvi.02200-25-s0001.docx]

**Supplementary Figure for**

**DIDS modulates VDAC1 oligomerization to suppress intrinsic apoptosis and attenuates *in vitro* and *in vivo* RSV infection**

Siyu Lin, Xiaotong Chen, Meihua Luo, Xiaolu Cui, You Dai, Zhen Sun, Guikang Wang, Hong Peng, Ping Ling, Jinlin Long, Huifang Zhou, Changlei Luo, Yan-Fei Qi, Ke Zhang, Yu-Si Luo

**This file includes:**

Supplementary Figures 1


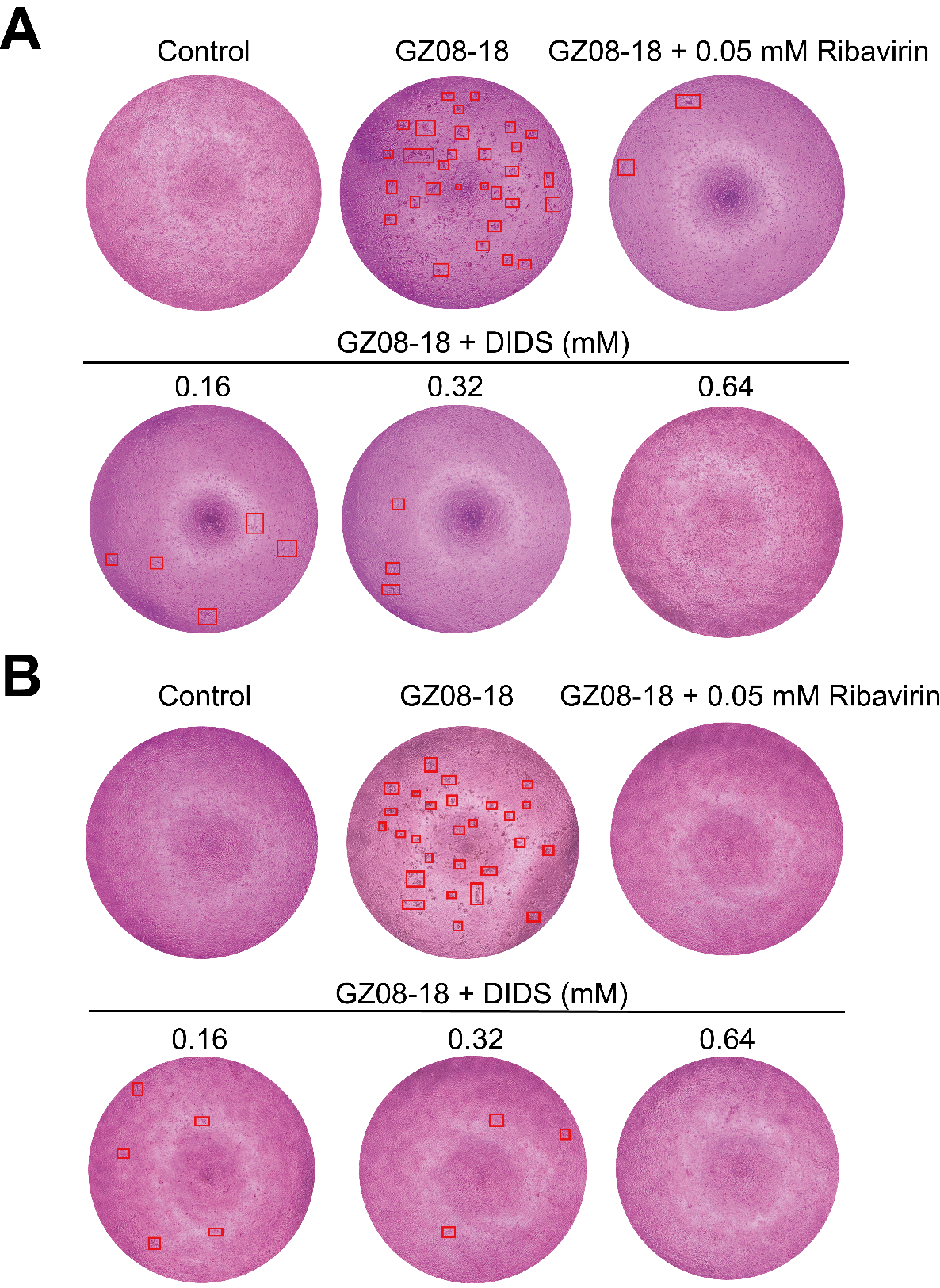


**Supplementary Figure 1. DIDS suppresses RSV-induced CPE in HEp-2 (A) and A549 (B) cells.**Cells were infected with 0.1 MOI GZ08-18 for 1 h, washed with sterilized PBS to remove unbound virus, and treated with DIDS at indicated concentrations. Controls included negative control (Control), virus control (GZ08-18), and positive control (GZ08-18 + 0.05 mM Ribavirin). Representative images of RSV-induced CPE, highlighted by red boxes, were captured at 48 hpi (40 x magnification).
