# Supplementary material for: DIDS modulates VDAC1 oligomerization to suppress intrinsic apoptosis and attenuates in vitro and in vivo RSV infection
Source: J Virol. 2026 Feb 11;100(3):e02200-25. doi: 10.1128/jvi.02200-25 (PMC13011466; doi:10.1128/jvi.02200-25)
Supplement: Fig. S2 — DIDS does not cause RAD51 expression change upon RSV infection. [file jvi.02200-25-s0002.docx]

**Supplementary Figure for**

**DIDS modulates VDAC1 oligomerization to suppress intrinsic apoptosis and attenuates *in vitro* and *in vivo* RSV infection**

Siyu Lin, Xiaotong Chen, Meihua Luo, Xiaolu Cui, You Dai, Zhen Sun, Guikang Wang, Hong Peng, Ping Ling, Jinlin Long, Huifang Zhou, Changlei Luo, Yan-Fei Qi, Ke Zhang, Yu-Si Luo

**This file includes:**

Supplementary Figures 2


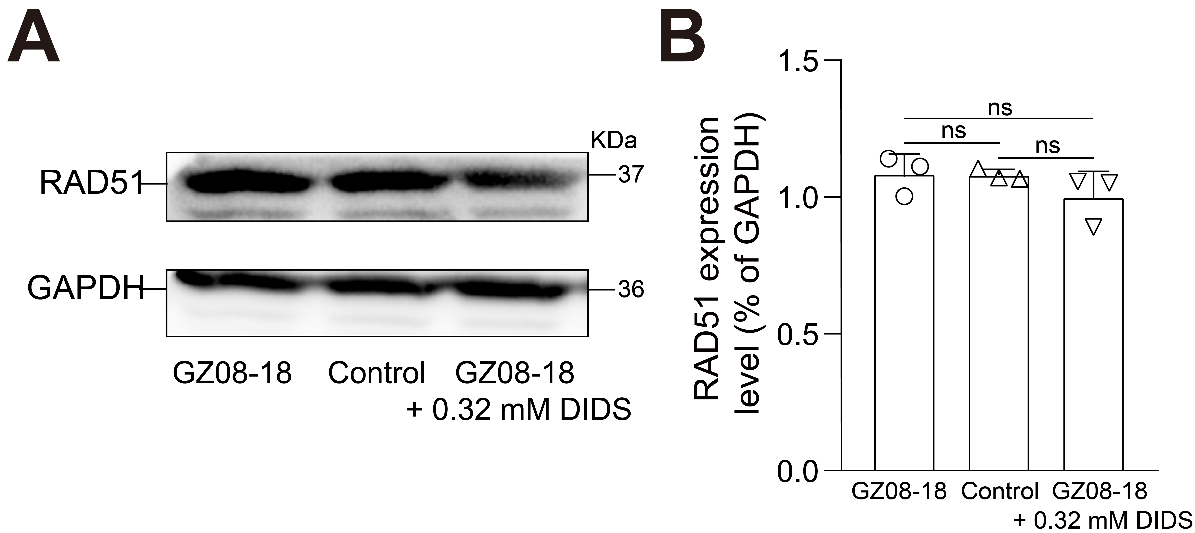
 **Supplementary Figure 2. DIDS dose not cause RAD51 expression change upon RSV infection.** (**A**) Western blotting (WB) result of RAD51 expression level from groups of GZ08-18, Control, and GZ08-18 + 0.32 mM DIDS. HEp-2 cells were infected with 0.1 MOI GZ08-18 for 1 h. For DIDS administration group, the supernatant was removed and replaced with medium containing 0.32 mM DIDS. RAD51 protein expression was assessed by WB at 48 hpi, with GAPDH serving as a loading control. (**B**) Quantification of RAD51 expression using ImageJ (v1.53i; National Institutes of Health, USA). Data were presented as mean + SD (***n*** = 3 per group). ns: non-statistical.
