# Supplementary material for: DIDS modulates VDAC1 oligomerization to suppress intrinsic apoptosis and attenuates in vitro and in vivo RSV infection
Source: J Virol. 2026 Feb 11;100(3):e02200-25. doi: 10.1128/jvi.02200-25 (PMC13011466; doi:10.1128/jvi.02200-25)
Supplement: Fig. S3 — Verification of siRNA-mediated VDAC1 knockdown. [file jvi.02200-25-s0003.docx]

**Supplementary Figure for**

**DIDS modulates VDAC1 oligomerization to suppress intrinsic apoptosis and attenuates *in vitro* and *in vivo* RSV infection**

Siyu Lin, Xiaotong Chen, Meihua Luo, Xiaolu Cui, You Dai, Zhen Sun, Guikang Wang, Hong Peng, Ping Ling, Jinlin Long, Huifang Zhou, Changlei Luo, Yan-Fei Qi, Ke Zhang, Yu-Si Luo

**This file includes:**

Supplementary Figures 3


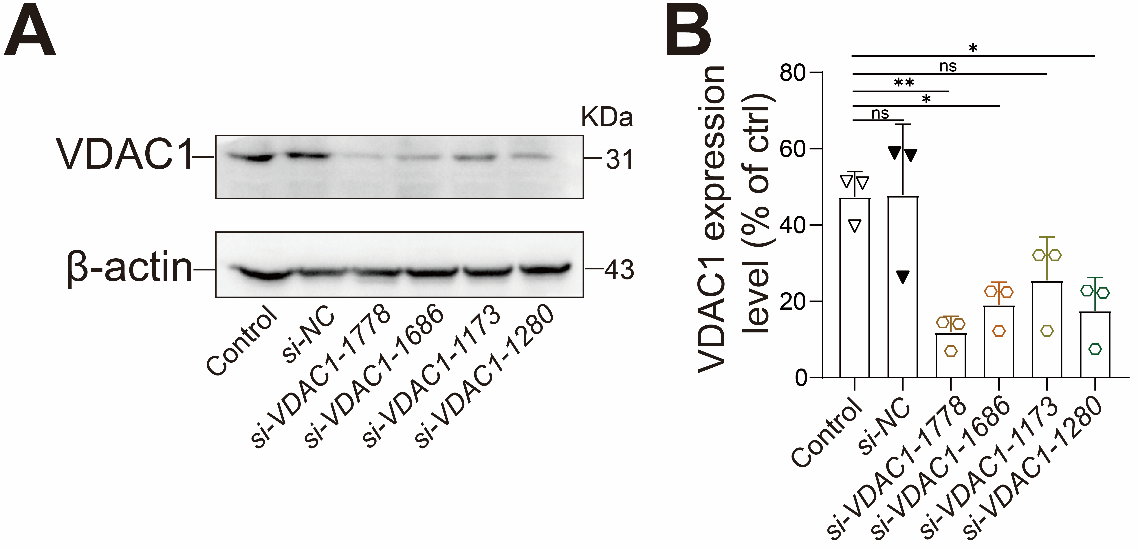


**Supplementary Figure 3. The *siRNA*-mediated VDAC1 knockdown is verified.** (**A**) WB result of VDAC1 expression level from groups of Control, *si-NC*, *si-VDAC1-1778*, *si-VDAC1-1686*, *si-VDAC1-1173*, and *si-VDAC1-1280*. HEp-2 cells were transfected with 80 nM of four siRNAs targeting VDAC1 (*si-VDAC1-1778*, *-1686*, *-1173*, *-1280*) or a negative control siRNA (*si-NC*). VDAC1 protein expression was assessed by WB 48 h post-transfection, with β-actin serving as a loading control. (**B**) Quantification of VDAC1 expression using ImageJ (v1.53i; National Institutes of Health, USA). Data were presented as mean + SD (***n*** = 3 per group). *p < 0.05, **p < 0.01, ns: non-statistical.
