# Supplementary material for: DIDS modulates VDAC1 oligomerization to suppress intrinsic apoptosis and attenuates in vitro and in vivo RSV infection
Source: J Virol. 2026 Feb 11;100(3):e02200-25. doi: 10.1128/jvi.02200-25 (PMC13011466; doi:10.1128/jvi.02200-25)
Supplement: Fig. S4 — DIDS does not cause intracellular Ca2+ influx upon RSV infection. [file jvi.02200-25-s0004.docx]

**Supplementary Figure for**

**DIDS modulates VDAC1 oligomerization to suppress intrinsic apoptosis and attenuates *in vitro* and *in vivo* RSV infection**

Siyu Lin, Xiaotong Chen, Meihua Luo, Xiaolu Cui, You Dai, Zhen Sun, Guikang Wang, Hong Peng, Ping Ling, Jinlin Long, Huifang Zhou, Changlei Luo, Yan-Fei Qi, Ke Zhang, Yu-Si Luo

**This file includes:**

Supplementary Figures 4


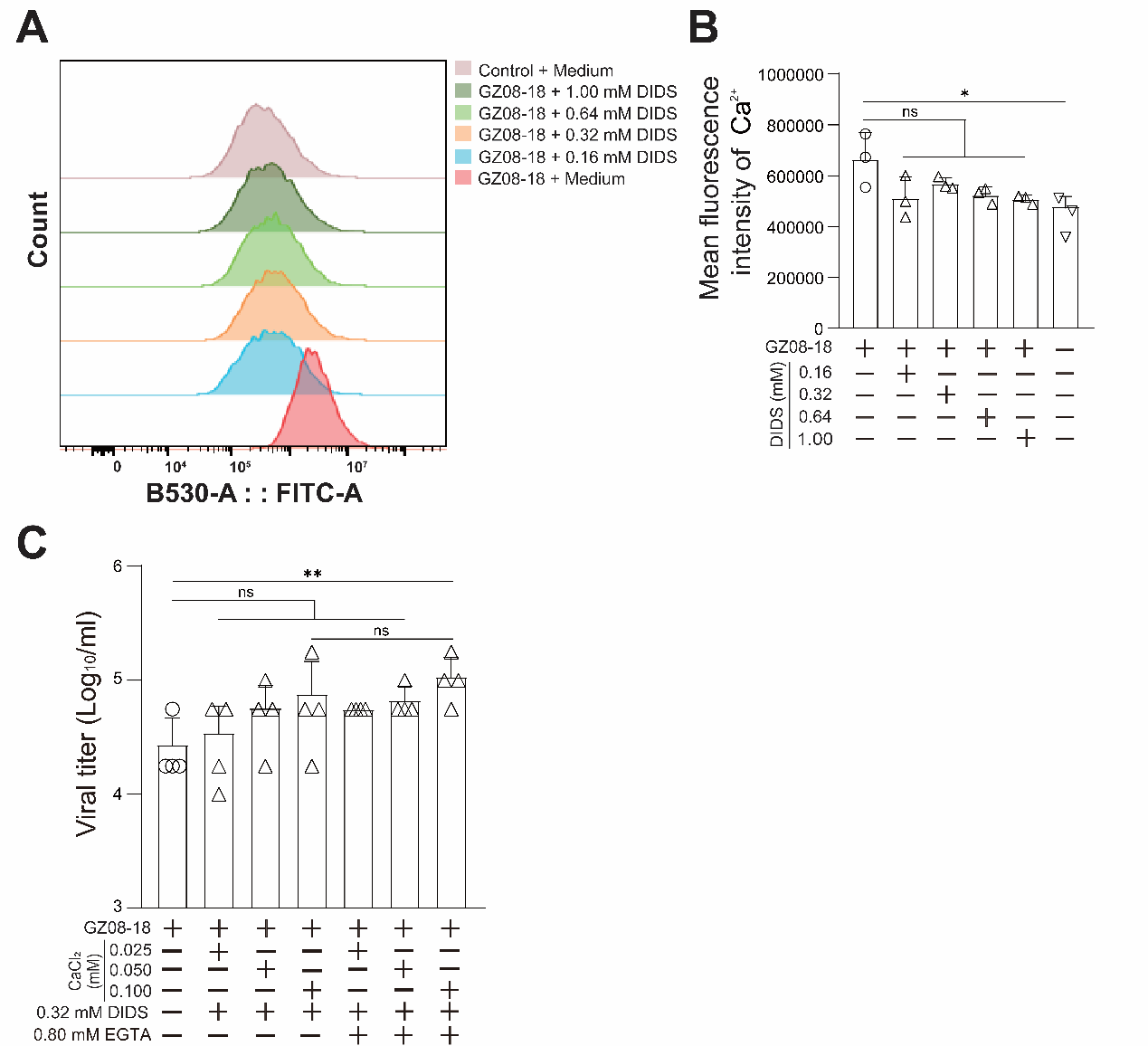


**Supplementary Figure 4. DIDS dose not cause intracellular Ca^2+^ influx upon RSV infection.** (**A**) Flow diagram of intracellular Ca^2+^ fluorescence intensity from groups of Control + Medium, GZ08-18 + 1.00 mM DIDS, GZ08-18 + 0.64 mM DIDS, GZ08-18 + 0.32 mM DIDS, GZ08-18 + 0.16 mM DIDS, and GZ08-18 + Medium. HEp-2 cells were infected with RSV at a MOI of 0.1 for 1 h. The supernatant was removed and replaced with medium containing 1.00, 0.64, 0.32, and 0.16 mM DIDS. At 48 hpi, cell supernatants were collected, and intracellular Ca^2+^ levels were measured by flow cytometry (CytoFlex3, Beckman Coulter, Inc., Brea, CA, USA) using 5 μM Fluo-8 AM (MX4505, Shanghai Maokang Co., Shanghai, China). (**B**) Quantification of mean fluorescence intensity for intracellular Ca^2+^ in groups of GZ08-18 + Medium, GZ08-18 + 0.16 mM DIDS, GZ08-18 + 0.32 mM DIDS, GZ08-18 + 0.64 mM DIDS, GZ08-18 + 1.00 mM DIDS, and Control + Medium. (**C**) Viral titer from groups of GZ08-18 + Medium, GZ08-18 + 0.025 mM CaCl_2_ + 0.32 mM DIDS, GZ08-18 + 0.050 mM CaCl_2_ + 0.32 mM DIDS, and GZ08-18 + 0.100 mM CaCl_2_ + 0.32 mM DIDS, or GZ08-18 + 0.025 mM CaCl_2_ + 0.32 mM DIDS + 0.80 mM EGTA, GZ08-18 + 0.050 mM CaCl_2_ + 0.32 mM DIDS + 0.80 mM EGTA, and GZ08-18 + 0.100 mM CaCl_2_ + 0.32 mM DIDS + 0.80 mM EGTA. HEp-2 cells were infected with GZ08-18 at a MOI of 0.1 for 1 h. Then the culture medium was replaced with fresh media containing 0.025, 0.050, and 0.100 mM CaCl_2_ plus 0.32 mM DIDS, or 0.025, 0.050, and 0.100 mM CaCl_2_ plus 0.32 mM DIDS and 0.80 mM EGTA, respectively. TCID_50_ assay was performed to detect RSV titer in supernatant samples at 48 hpi. Data were presented as mean + SD (***n*** = 3 or 4 per group). *p < 0.05 and **p < 0.01, ns: non-statistical.
