# Supplementary material for: DIDS modulates VDAC1 oligomerization to suppress intrinsic apoptosis and attenuates in vitro and in vivo RSV infection
Source: J Virol. 2026 Feb 11;100(3):e02200-25. doi: 10.1128/jvi.02200-25 (PMC13011466; doi:10.1128/jvi.02200-25)
Supplement: Table S1 — siRNA sequences of this study. [file jvi.02200-25-s0005.docx]

**Supplementary Table for**

**DIDS modulates VDAC1 oligomerization to suppress intrinsic apoptosis and attenuates *in vitro* and *in vivo* RSV infection**

Siyu Lin, Xiaotong Chen, Meihua Luo, Xiaolu Cui, You Dai, Zhen Sun, Guikang Wang, Hong Peng, Ping Ling, Jinlin Long, Huifang Zhou, Changlei Luo, Yan-Fei Qi, Ke Zhang, Yu-Si Luo

**This file includes:**

Supplementary Table 1

**Supplementary Table 1.** ***si-RNA* sequences of this study**

| *si-RNA*s | Sequences (5’→3’) | Orientation |
| --- | --- | --- |
| *si-VDAC1-1686* | CGGCUCCAUUUACCAGAAATT | Forward |
|  | UUUCUGGUAAAUGGAGCCGTT | Reverse |
| *si-VDAC1-1778* | CAGCCAAGUAUCAGAUUGATT | Forward |
|  | UCAAUCUGAUACUUGGCUGTT | Reverse |
| *si-VDAC1-1173* | GGGCUAUGGAUUUGGCUUATT | Forward |
|  | UAAGCCAAAUCCAUAGCCCTT | Reverse |
| *si-VDAC1-1280* | GCAGUCUGGAAACCAAGUATT | Forward |
|  | UACUUGGUUUCCAGACUGCTT | Reverse |
| *si-NC* | UUCUCCGAACGUGUCACGUTT | Forward |
|  | ACGUGACACGUUCGGAGAATT | Reverse |
